# Translational Development of Oral FXIa Inhibitors: A Systematic Review of Molecular Design, Pharmacology, and Indication-Specific Clinical Evidence

**DOI:** 10.3390/ph19081298

**Published:** 2026-08-17

**Authors:** Michał Janiak, Katarzyna Mądra-Gackowska, Lidia Wydeheft, Marcin Gackowski

**Affiliations:** 1 Department of Toxicology and Bromatology, Faculty of Pharmacy, Ludwik Rydygier Collegium Medicum in Bydgoszcz, Nicolaus Copernicus University in Torun, Jurasza 2 Str., 85-089 Bydgoszcz, Poland; michal.janiak@doktorant.umk.pl; 2 Doctoral School of Medical and Health Sciences, Ludwik Rydygier Collegium Medicum, Nicolaus Copernicus University in Toruń, Jagiellońska 13-15 Str., 85-067 Bydgoszcz, Poland; 3 Department of Geriatrics, Faculty of Health Sciences, L. Rydygier Collegium Medicum in Bydgoszcz, Nicolaus Copernicus University in Torun, Skłodowskiej Curie 9 Str., 85-094 Bydgoszcz, Poland; katarzyna.madra@cm.umk.pl (K.M.-G.); lidia.wydeheft@gmail.com (L.W.)

**Keywords:** factor XIa inhibitor, oral anticoagulant, milvexian, asundexian, thrombosis, bleeding, pharmacokinetics/pharmacodynamics, systematic review

## Abstract

**Background/Objectives:** Oral small-molecule inhibitors of activated factor XI (FXIa) are intended to attenuate thrombosis with less disruption of hemostasis than established anticoagulants, but clinical outcomes have not consistently paralleled pharmacodynamic target engagement. This systematic review integrated molecular design, preclinical pharmacology, human pharmacokinetics/pharmacodynamics (PK/PD), and clinical outcomes to identify where translation is maintained or lost. **Methods:** The review followed PRISMA 2020 and PRISMA-S and was registered in PROSPERO (CRD420261356169). PubMed/MEDLINE, Scopus, and Web of Science Core Collection were searched from inception through 30 March 2026 and rerun on 11 August 2026, with a final publication-eligibility cut-off of 30 June 2026. Owing to heterogeneity, evidence was synthesized narratively. **Results:** Sixty-eight eligible reports were included, comprising seven randomized parent outcome trials and nine reports with in vivo thrombosis or bleeding experiments. In the OCEANIC-STROKE trial, ischemic stroke occurred in 6.2% with asundexian versus 8.4% with placebo (HR 0.74, 95% CI 0.65–0.84), while major bleeding was 1.9% versus 1.7% (HR 1.10, 95% CI 0.85–1.44). In OCEANIC-AF, stroke or systemic embolism occurred in 1.3% with asundexian versus 0.4% with apixaban (HR 3.79, 95% CI 2.46–5.83), despite less major bleeding (0.2% vs. 0.7%; HR 0.32, 95% CI 0.18–0.55). **Conclusions:** Oral small-molecule FXIa inhibition is pharmacologically coherent, but clinical benefit is indication-, comparator-, and background-therapy-dependent. PD markers confirm target engagement but are not validated efficacy surrogates; hard clinical outcomes remain decisive.

## 1. Introduction

Venous thromboembolism (VTE) and atrial fibrillation (AF) are major indications for anticoagulation. The incidence of diagnosed VTE is approximately 1–2 per 1000 people per year and rises substantially with age [1]. AF likewise imposes a large and increasing global burden [2] and is strongly associated with ischemic stroke [3]. Oral anticoagulation is therefore central to prevention and treatment of thromboembolic disease.

Current therapy relies on vitamin K antagonists, heparins, and direct oral anticoagulants (DOACs) targeting thrombin or factor Xa. DOACs provide more predictable pharmacokinetics and, in AF, reduce intracranial hemorrhage compared with warfarin, but clinically important bleeding remains a major limitation [4,5]. This problem is particularly relevant in older adults, patients with renal dysfunction, and those receiving concomitant antiplatelet or interacting therapies [4,6,7]. These limitations motivate the search for anticoagulant targets with a wider therapeutic margin.

Factor XI (FXI) and its activated form FXIa are attractive targets because the intrinsic/contact pathway contributes to amplification of thrombin generation and stabilization of pathologic thrombi, whereas congenital FXI deficiency is generally associated with a milder bleeding phenotype than deficiencies of factor VIII or IX [4,8]. Epidemiological and experimental observations therefore support the possibility that reducing FXI/FXIa activity could attenuate thrombosis with less disruption of physiological hemostasis. However, the frequently invoked ‘separation of thrombosis from hemostasis’ remains a therapeutic hypothesis that requires indication-specific clinical validation rather than a proven class effect [4,8].

Several therapeutic modalities target FXI or FXIa. Oral small-molecule direct FXIa inhibitors include milvexian, asundexian, ONO-7684, and SHR2285, whereas BMS-962212 and frunexian represent parenteral small-molecule approaches [9,10,11,12,13,14,15,16,17,18,19]. Monoclonal antibodies and other biologic strategies provide longer-acting parenteral inhibition [20,21,22,23,24], while antisense and RNA-based approaches reduce hepatic FXI synthesis [25,26]. In parallel, allosteric, heparin-mimetic, and sulfated small-molecule scaffolds broaden the discovery-stage chemical space [27,28]. The primary systematic evidence base of this review was restricted to oral small-molecule direct FXIa inhibitors and discovery-stage small-molecule series relevant to oral anticoagulant development; other modalities were considered only as contextual comparators where appropriate.

Preclinical programs for asundexian and milvexian established potent and selective FXIa inhibition, intrinsic-pathway pharmacodynamic effects, and antithrombotic activity in rabbit models with limited effects on the bleeding endpoints used in those experiments [9,10,11,12,13]. First-in-human and phase I studies subsequently showed predictable oral PK/PD and dose- or exposure-related target engagement for asundexian, milvexian, ONO-7684, and SHR2285 [14,15,16,17,18,29,30]. Clinical outcome trials, however, produced divergent results. Milvexian showed a positive postoperative VTE signal after knee arthroplasty [31], while PACIFIC-Stroke, AXIOMATIC-SSP, and PACIFIC-AMI did not establish significant primary efficacy benefits [32,33,34]. OCEANIC-AF demonstrated inferior prevention of stroke or systemic embolism with asundexian versus apixaban despite less major bleeding [35]. In contrast, the phase III OCEANIC-STROKE trial showed that asundexian added to antiplatelet therapy reduced recurrent ischemic stroke after noncardioembolic ischemic stroke or high-risk TIA without a statistically significant increase in major bleeding [36]. These contrasting phase III results make the relationship between target engagement, thrombotic mechanism, comparator, background therapy, and clinical benefit a central translational question.

Recent reviews address different questions. Prakash et al. provided a broad narrative overview of FXI/FXIa inhibitors across modalities and thromboembolic settings [8]. Xue et al. systematically compared FXI/FXIa inhibitors with direct oral anticoagulants specifically in atrial fibrillation [37], whereas Steiner et al. focused on patients with end-stage kidney disease receiving hemodialysis and included multiple inhibitor modalities [38]. In contrast, the present systematic review uses an oral small-molecule focus, a publication-eligibility cut-off of 30 June 2026, and a cross-stage framework that integrates medicinal chemistry, in vitro and animal pharmacology, ADMET/DMPK, human PK/PD, and indication-specific clinical outcomes. This design is intended to locate translational breakpoints rather than to provide another broad class overview or a single-population efficacy comparison.

Accordingly, the review question was structured around four linked elements: populations ranging from healthy volunteers to patients with thromboembolic disease; oral small-molecule FXIa inhibitors as the intervention; placebo, active anticoagulants, background therapy, or baseline conditions as appropriate comparators; and pharmacodynamic target engagement, antithrombotic efficacy, and bleeding as the principal outcomes. The primary objective was to determine how molecular design, preclinical pharmacology, and human PK/PD translate into efficacy and bleeding outcomes across clinical indications and to identify factors that may explain discordance between pharmacodynamic target engagement and clinical benefit.

## 2. Materials and Methods

### 2.1. Study Design, Registration, and Scope

This systematic review was conducted and reported in accordance with the PRISMA 2020 statement and the PRISMA-S extension for reporting literature searches in systematic reviews [39,40]. The completed PRISMA 2020 checklist is provided in Supplementary Table S1. The review was registered in PROSPERO under registration number CRD420261356169. Registration occurred after pilot work and initial searching/screening had begun; however, the core review question, databases, eligibility framework, and evidence domains remained focused on the translational development of oral small-molecule FXIa inhibitors. The bibliographic searches were subsequently rerun to improve currency and retrieval sensitivity without changing the final operational eligibility criteria. The full internal review protocol was not made publicly available; the PROSPERO record and Supplementary Tables S1 and S2 provide the publicly accessible registration and reporting details.

The review synthesized evidence across the development pathway of oral small-molecule FXIa inhibitors, including molecular design, in vitro pharmacology, preclinical in vivo pharmacology, PK/PD, clinical efficacy, and bleeding safety. The primary evidence base was restricted to oral small-molecule direct FXIa inhibitors and discovery-stage small-molecule FXIa inhibitor series relevant to oral anticoagulant development. Non-small-molecule FXI/FXIa-targeting modalities, including monoclonal antibodies, antisense oligonucleotides, small interfering RNA, and other biologic or nucleic acid-based therapies, were excluded from the systematic evidence base. When mentioned, these modalities were treated only as contextual comparators and were not counted among included studies.

### 2.2. Information Sources, Search Strategy, and Study Selection

The initial database searches were conducted in PubMed/MEDLINE, Scopus, and Web of Science Core Collection, including the Science Citation Index, from database inception through 30 March 2026. Searches were restricted to English-language publications. The searches were rerun on 11 August 2026 to identify additional eligible publications, with a final publication-eligibility cut-off of 30 June 2026. The rerun used the same core concepts in all three bibliographic databases and was supplemented by compound-specific searches and citation searching to improve sensitivity. Some technical exports covered a broader date range than the final eligibility window; records published after 30 June 2026 were excluded during eligibility assessment. Clinical trial registers, including ClinicalTrials.gov and the EU Clinical Trials Register where applicable, were searched to identify ongoing, completed, or unpublished clinical studies. Reference lists of included studies and relevant reviews were screened manually, and forward-citation searching was used to identify additional potentially eligible records.

**Figure 1:**
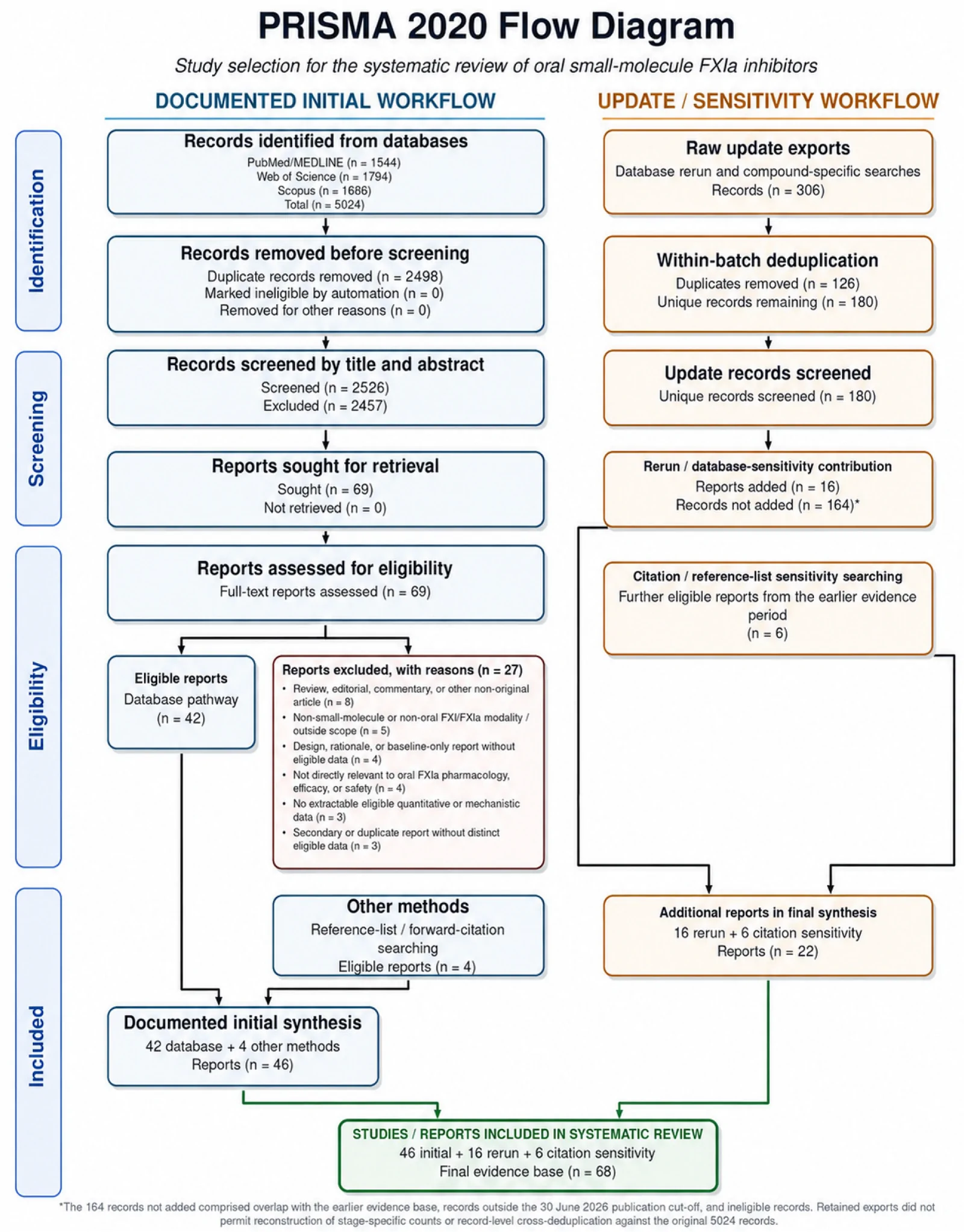
Adapted PRISMA 2020 study-selection flow for the documented initial workflow and the separately reported update/sensitivity pathway. The pathways are shown separately because retained exports did not permit record-level cross-deduplication against the original 5024 records. The 164 update records not added to the synthesis comprised overlap with the earlier evidence base, records outside the 30 June 2026 cut-off, and ineligible records; stage-specific counts could not be reconstructed. The final evidence base contained 68 eligible reports.

Within the documented initial workflow, reference-list and forward-citation searching of the SHR2285 and milvexian clinical pharmacology programs identified four eligible reports in addition to those identified through bibliographic database searching: three oral phase I reports of SHR2285 and one oral milvexian renal-impairment pharmacokinetic report. These reports were represented in the PRISMA 2020 ‘other methods’ pathway. The later rerun/sensitivity workflow was kept separate in Figure 1 because retained original exports did not permit reliable record-level cross-deduplication against the original 5024 records.

The implemented database searches used free-text terms adapted to database-specific syntax; no additional controlled-vocabulary terms were applied. The core search concept was based on the PROSPERO-defined strategy:

TITLE-ABS-KEY (“factor XI” OR “factor XIa” OR FXI OR FXIa OR “activated factor XI”) AND (inhibitor* OR “small molecule*” OR “oral” OR “drug design”).

Because TITLE-ABS-KEY is Scopus-specific syntax, equivalent title/abstract or topic-field syntax was applied in PubMed/MEDLINE and Web of Science. Full database-specific initial and rerun strategies, actual rerun date, technical export windows, final publication-eligibility cut-off, and database yields are provided in Supplementary Table S2.

The documented initial database searches identified 5024 records: PubMed/MEDLINE, *n* = 1544; Web of Science, *n* = 1794; and Scopus, *n* = 1686. After removal of 2498 duplicate records, 2526 records underwent title and abstract screening and 2457 were excluded. Sixty-nine reports were sought for retrieval and assessed for eligibility; none were unavailable. Twenty-seven reports were excluded with recorded reasons, leaving 42 eligible reports from the database pathway. Reference-list and forward-citation searching contributed four further eligible reports, giving 46 reports in the documented initial synthesis. The rerun and compound-specific database exports yielded 306 raw records; within this update/sensitivity batch, 126 duplicates were removed and 180 unique records were screened. Sixteen reports from this database/sensitivity set were added to the primary synthesis. Citation/reference-list sensitivity searching identified six further eligible reports from the earlier evidence period that were not represented in the original synthesis. The final systematic synthesis therefore comprised 68 eligible reports. Because record-level overlap between the rerun batch and the original 5024 records could not be reconstructed from retained exports, the two identification pathways are shown separately in Figure 1 rather than being retrospectively merged.

### 2.3. Eligibility Criteria and Data Extraction

Studies were eligible if they reported original quantitative or mechanistic data on orally administered small-molecule FXIa inhibitors. Discovery-stage series were eligible only when the report provided evidence relevant to oral candidate optimization, such as explicit oral administration or exposure, permeability, metabolic stability, DMPK/developability data, or an in vivo program linked to oral development. Exploratory hybrid or allosteric series were included only when they presented original quantitative FXIa-focused small-molecule data with a stated translational rationale; otherwise, they were treated as context-only evidence. Eligible domains included medicinal chemistry, structure-based design, structure–activity relationships, in vitro enzymatic or plasma pharmacology, selectivity, ADMET/DMPK, animal thrombosis or bleeding models, phase I PK/PD and clinical pharmacology studies, and phase II/III efficacy or safety trials.

Clinical studies were eligible if they evaluated oral small-molecule FXIa inhibitors in healthy volunteers or patients with thromboembolic conditions, including AF, VTE, postoperative thromboprophylaxis, acute coronary syndromes (ACS), or secondary stroke prevention. Both comparative and non-comparative studies were eligible if they provided original data relevant to FXIa inhibition.

Studies were excluded if they investigated non-small-molecule FXI/FXIa-targeting modalities, non-oral interventions unless oral administration was part of the investigated intervention, indirect or non-selective anticoagulant mechanisms unrelated to direct FXIa inhibition, purely biological FXI/FXIa studies without an inhibitor or therapeutic candidate, reviews, meta-analyses, editorials, commentaries, letters, conference abstracts without accessible full data, case reports or case series without quantitative data, or publications without extractable original data.

Reports without clinical outcome or eligible pharmacological data, including design, rationale, and baseline characteristics reports, were not treated as primary efficacy or safety evidence but could be discussed as contextual evidence for ongoing development programs. Secondary and subgroup analyses were eligible only when they provided distinct data relevant to the review question and were not counted as independent parent trials when derived from the same clinical program. Context-only records and excluded evidence types are summarized in Supplementary Table S4.

Initial title/abstract screening and primary data extraction were performed by M.J. using predefined eligibility and extraction templates. M.G. independently reassessed the eligibility of the 46 reports included in the documented initial synthesis and all 27 reports excluded after full-text assessment, and independently checked the key extracted data used in the tables and narrative synthesis against the source publications. Disagreements or uncertainties in that workflow were resolved by consensus after reassessment of the relevant full text. The additional reports identified through the rerun and sensitivity searches were screened and extracted using the same predefined eligibility and extraction framework. This targeted verification did not constitute duplicate screening of all 2526 original title/abstract records or duplicate extraction of every variable. Study authors were not contacted for additional information.

Extracted variables included compound name, development code, molecular target, route of administration, study design, experimental system or clinical population, comparator, dose and regimen, potency metrics, selectivity profile, PD markers, ADMET/DMPK parameters, in vivo thrombosis and bleeding endpoints, clinical efficacy outcomes, safety outcomes, and background antithrombotic therapy where applicable. The main extracted outcomes included enzymatic potency, selectivity, plasma-based PD effects, PK/DMPK parameters, in vivo antithrombotic and bleeding endpoints, clinical efficacy outcomes, bleeding outcomes, adverse events, and treatment discontinuations.

### 2.4. Methodological-Quality Assessment

Methodological quality was assessed according to study type and evidence domain. Randomized clinical outcome trials were assessed with the Cochrane Risk of Bias 2 (RoB 2) tool for the principal efficacy and/or safety outcome emphasized in this review, covering bias arising from the randomization process, deviations from intended interventions, missing outcome data, measurement of the outcome, and selection of the reported result. Animal studies reporting in vivo thrombosis or bleeding experiments were assessed with the SYRCLE risk-of-bias tool, covering sequence generation, baseline comparability, allocation concealment, random housing, blinding, random outcome assessment, incomplete outcome data, selective reporting, and other potential sources of bias. Two reviewers (M.J. and M.G.) independently assigned domain-level judgments and resolved disagreements by consensus.

Phase I, clinical pharmacology, in vitro, medicinal chemistry, SAR, computational, and ADMET/DMPK studies were appraised descriptively because RoB 2 and SYRCLE are not applicable to these designs. The appraisal considered assay transparency, FXI/FXIa source, comparator reporting, numerical potency and selectivity, plasma-based PD readouts, ADMET/DMPK conditions, and agreement between computational predictions and experimental validation. Formal study-level judgments were assigned only to randomized clinical outcome trials and animal in vivo studies (Supplementary Table S5B,C); the remaining domains were appraised at the evidence-domain level using the framework in Supplementary Table S5A, not graded individually for risk of bias. No study was excluded on methodological-quality grounds. Reporting bias and certainty of evidence were not formally assessed, consistent with the registered protocol.

### 2.5. Data Synthesis and Protocol Considerations

Because of substantial heterogeneity in compound series, assay systems, animal models, clinical populations, PD markers, comparators, endpoint definitions, and reporting formats, quantitative meta-analysis was not performed. Evidence was synthesized narratively using a modular translational framework.

Findings were grouped into discovery and medicinal chemistry, in vitro pharmacology, ADMET/DMPK and clinical pharmacology, in vivo thrombosis and bleeding models, and clinical efficacy and safety domains. Cross-domain synthesis focused on relationships between molecular design, potency, selectivity, PD, oral exposure, DMPK, in vivo antithrombotic and bleeding endpoints, human PK/PD, clinical efficacy, and bleeding outcomes. Studies contributing to more than one evidence domain were counted once but synthesized across all relevant domains.

To examine dose–marker–outcome relationships without imposing invalid cross-trial equivalence, clinical reports were organized in a structured dose/exposure–pharmacodynamic–outcome matrix (Supplementary Table S7). A pooled or three-dimensional quantitative model was not undertaken because compounds, assays, sampling times, clinical indications, comparators, and endpoint definitions were not sufficiently commensurable. Rabbit thrombosis and bleeding studies were standardized descriptively by model, induction method, antithrombotic endpoint, bleeding endpoint, timing, and comparator (Supplementary Table S8); numerical normalization across studies was not performed because thrombus weight, carotid blood flow, bleeding time, and blood loss measure different constructs under non-equivalent protocols.

Context-only sources, including reviews, non-small-molecule FXI/FXIa modalities, and trial design records without eligible outcome or pharmacological data, were not counted among the 68 included reports. Secondary or subgroup reports were counted as eligible reports only when they contributed distinct data relevant to the review question and were not treated as independent parent trials. Design, rationale, or baseline reports were used only to contextualize development programs and were not interpreted as evidence of established efficacy or safety.

The review was registered after pilot work and initial searching/screening had begun. The documented initial title/abstract screen and primary extraction were performed by one reviewer, followed by independent verification of the 46 initially included reports, all 27 original full-text exclusions, and key extracted data by a second reviewer; the complete title/abstract set was not independently rescreened and every extracted variable was not independently re-extracted. The later rerun/sensitivity workflow used the same eligibility criteria and final evidence domains. The publication-eligibility window was extended from 30 March to 30 June 2026, while the actual rerun was performed on 11 August 2026. These review procedures differed from the registered plan for independent screening and data extraction by at least two reviewers and are reported as protocol deviations.

## 3. Results

### 3.1. Study Selection

The documented initial database searches identified 5024 records across PubMed/MEDLINE (*n* = 1544), Web of Science (*n* = 1794), and Scopus (*n* = 1686). After removal of 2498 duplicate records, 2526 records remained for title and abstract screening.

During title and abstract screening, 2457 records were excluded because they did not meet the predefined eligibility criteria. The main reasons for exclusion at this stage were records outside the oral small-molecule FXIa inhibitor scope, studies focused on other coagulation or contact-pathway targets, publications addressing FXI/FXIa only as a biomarker, genetic factor, deficiency state, or pathophysiological variable, review articles, editorials or commentaries, conference abstracts without accessible full data, case reports, protocol or design papers without extractable eligible data, and studies evaluating modalities outside the predefined synthesis scope.

After title and abstract screening, 69 reports were sought for retrieval and assessed for eligibility. No reports were unavailable for retrieval. Twenty-seven reports were excluded after eligibility assessment. The reasons for exclusion were: review, editorial, commentary, or other non-original article, *n* = 8; non-small-molecule or non-oral FXI/FXIa-targeting modality or outside review scope, *n* = 5; design, rationale, or baseline-only article without eligible outcome or pharmacology data, *n* = 4; not directly relevant to oral FXIa inhibitor pharmacology, efficacy, or safety, *n* = 4; no extractable eligible quantitative or mechanistic data, *n* = 3; and secondary or duplicate report without distinct eligible data, *n* = 3.

The documented initial database pathway therefore contributed 42 eligible reports, and reference-list/forward-citation searching contributed four further eligible reports, giving 46 reports in the initial synthesis. The later rerun and compound-specific database exports yielded 306 raw records; 126 within-update duplicates were removed, leaving 180 unique records for screening. Sixteen reports from this rerun/database-sensitivity set were added to the synthesis. Six additional eligible reports were identified by citation/reference-list sensitivity searching across the earlier evidence period.

The final systematic evidence base comprised 68 eligible full-text studies/reports. The added evidence included clinical pharmacology and PK/PD reports, secondary analyses with distinct data, laboratory and computational studies, optimized medicinal chemistry/preclinical series, and the phase III OCEANIC-STROKE outcome trial. Studies contributing to more than one evidence domain were counted once but synthesized across all relevant domains.

The study-selection process, including the documented initial workflow and the separately reported rerun/sensitivity pathway, is summarized in the PRISMA 2020 flow diagram.

### 3.2. General Characteristics of Included Studies

The final systematic evidence base comprised 68 eligible full-text studies/reports that met the predefined eligibility criteria. Consistent with the PROSPERO-aligned scope, the included reports covered the translational development pathway of oral small-molecule FXIa inhibitors across computational, medicinal chemistry, in vitro, preclinical, clinical pharmacology, secondary clinical analysis, and clinical outcome domains. Their characteristics are summarized in Supplementary Table S6.

To improve interpretability of this heterogeneous evidence base, the included studies were additionally organized according to their translational role rather than only by study design or compound name. This framework separated clinically developed oral FXIa inhibitors from human phase I candidates, optimized preclinical small-molecule series, discovery-stage active-site scaffolds, exploratory hybrid or allosteric scaffolds, and laboratory studies addressing assay interference or neutralization. The resulting evidence matrix is presented in Table 1 and is intended to clarify the level of translational maturity represented by each compound or scaffold group.

Consistent with this matrix, the evidence base included studies of molecular design, medicinal chemistry, structure–activity relationships, enzymatic and plasma-based pharmacology, selectivity profiling, ADMET/DMPK characterization, in vivo thrombosis and bleeding models, phase I PK/PD studies, drug–drug interaction studies, special-population pharmacology studies, formulation and food-effect studies, and phase II/III clinical outcome studies. Studies contributing to more than one evidence domain were counted once but synthesized across all relevant domains.

The clinical and clinical pharmacology evidence primarily evaluated asundexian/BAY 2433334, milvexian/BMS-986177/JNJ-70033093, ONO-7684, and SHR2285. These studies included first-in-human trials, single- and multiple-dose phase I studies, ethnicity-specific pharmacology studies, intrinsic-factor studies, hepatic-impairment studies, drug–drug interaction studies, bioavailability and formulation studies, antiplatelet coadministration studies, and phase II or phase III clinical outcome studies in AF, noncardioembolic secondary stroke prevention, acute myocardial infarction (AMI), and postoperative VTE prevention. Clinical pharmacology studies were interpreted as evidence of exposure, target engagement, tolerability, and dose-development support, whereas clinical outcome trials were interpreted separately as evidence of indication-specific efficacy and bleeding safety.

The preclinical and translational evidence included medicinal chemistry, structure-based design, SAR optimization, molecular docking or molecular dynamics, enzymatic FXIa inhibition assays, selectivity profiling, ADMET/DMPK evaluation, plasma-based coagulation assays, laboratory assay interference studies, in vitro reversal or neutralization studies, and in vivo thrombosis or bleeding models. The main chemical or pharmacological categories included macrocyclic FXIa inhibitors, pyridine N-oxide derivatives, oxopyridine derivatives, triazole-based benzoic acid P2′ fragments, imidazole-carboxamide derivatives, pyrazole-carboxamide leads, allosteric small-molecule inhibitors, sulfonated non-saccharide molecules, and hybrid pyrroloquinolinone-based structures. Early scaffold-exploration studies were interpreted as discovery-stage or mechanistic evidence unless they also provided oral exposure, DMPK, in vivo, or clinical data supporting translational developability.

The experimental systems used across the included studies ranged from purified-enzyme assays and plasma-based coagulation assays to human plasma or whole-blood systems, ADMET/DMPK platforms, rabbit thrombosis models, bleeding-time or blood-loss models where reported, and human PK/PD or clinical outcome studies. This diversity allowed the evidence to be organized across successive stages of translational development: molecular optimization, functional anticoagulant activity, PK and PD characterization, in vivo antithrombotic and bleeding-liability assessment, and indication-specific clinical efficacy and safety.

Consistent with the predefined eligibility criteria, non-small-molecule FXI/FXIa-targeting strategies were not included in the 68-report systematic evidence base. Excluded modalities included monoclonal antibodies, antisense oligonucleotides, small interfering RNA, and other biologic or nucleic-acid-based interventions. Non-oral FXI/FXIa inhibitors were also excluded from the primary evidence base unless oral administration was part of the investigated intervention. Publications focused on these approaches were used only as contextual comparators where relevant and were not treated as primary evidence for the translational development of oral small-molecule FXIa inhibitors.

Trial design, rationale, and baseline characteristics reports without eligible outcome or pharmacological data were not treated as primary efficacy or safety evidence. When cited, these records were used only to contextualize ongoing or planned clinical development programs. Context-only records and excluded evidence types are summarized in Supplementary Table S4. This restriction preserved a focused and mechanistically coherent evidence base for evaluating oral small-molecule FXIa inhibitors while avoiding inappropriate extrapolation from non-oral, non-small-molecule, or context-only evidence.

### 3.3. Results of the Methodological-Quality Assessment

Seven randomized parent clinical outcome trials were assessed with RoB 2 for the principal outcomes emphasized in this review. PACIFIC-AF, PACIFIC-Stroke, AXIOMATIC-SSP, OCEANIC-AF, PACIFIC-AMI, and OCEANIC-STROKE were judged at low overall risk of bias. AXIOMATIC-TKR was judged at low risk for its primary venous thromboembolism outcome and as presenting some concerns for its principal bleeding outcome because assignment to milvexian versus enoxaparin was open-label, although outcomes were centrally adjudicated. Secondary analyses derived from these parent trials were not treated as additional independent RoB 2 units. Nine reports containing in vivo thrombosis or bleeding experiments were assessed with SYRCLE. Their overall risk of bias was judged unclear because reporting was generally insufficient for confident judgments on random sequence generation, allocation concealment, random housing, and blinding. Detailed judgments are presented in Supplementary Table S5B,C.

### 3.4. Preclinical, Medicinal Chemistry, and Translational Pharmacology Evidence

#### 3.4.1. Computational and Structure-Based Design

Computational and structure-guided approaches were frequently used in medicinal chemistry and preclinical discovery programs for small-molecule FXIa inhibitors, although the specific methods differed across chemical series. Protein structure-based or structure-aided design supported asundexian, fragment-derived active-site inhibitors, S2-subsite-directed inhibitors, pyridine N-oxide-based inhibitors, and newer S2′-focused optimized series [10,68,69,71,72,73,74]. Fragment-based lead generation, docking, molecular dynamics, and MM/GBSA calculations were used selectively for hit identification, binding-mode rationalization, subsite-directed optimization, and selectivity analysis [28,73,74,75,77,80,83]. A machine-learning QSAR analysis additionally modeled a curated set of approximately 3026 FXIa inhibitors to support virtual screening and rational design [79]. These computational approaches were interpreted as supportive discovery evidence and not as substitutes for experimental potency, developability, or in vivo validation.

The active-site subsite targeted during optimization differed across chemical series. Fragment-derived FXIa inhibitors were expanded from S1-binding fragments toward the S1′ and S2′ regions [74]. Triazole-based benzoic acid derivatives emphasized P2′/S2′ optimization while maintaining stable S1-pocket interactions [77]. S2-subsite-directed inhibitors were designed to gain additional S2 interactions and improve selectivity over plasma kallikrein [68]. Imidazole-carboxamide derivatives were modeled across the S1, S1′, and S2′ pockets [78], while pyridine N-oxide-based inhibitors were optimized mainly in the P1′ and P2′ regions using nonclassical interactions [69]. Together, these studies show that computational and structure-based approaches were used not as a single uniform workflow, but as complementary tools for hit identification, binding-mode rationalization, subsite-directed optimization, and selectivity improvement across different FXIa inhibitor chemotypes.

#### 3.4.2. Medicinal Chemistry and SAR of Small-Molecule FXIa Inhibitors

The included medicinal chemistry studies encompassed orthosteric active-site inhibitor series and a smaller set of allosteric programs. Active-site optimization targeted S1/S1′, S2, and P2′/S2′ regions depending on scaffold [68,69,70,74,75,76,77,78]. The asundexian program illustrated simultaneous optimization of potency, absorption, metabolic stability, CYP liability, and overall DMPK properties [10]. Additional orally bioavailable preclinical programs published within the final evidence window extended this framework: compound 43 combined high FXIa potency/selectivity with oral exposure and antithrombotic activity in mouse and rabbit thrombosis models [71], while F47 and FE12 represented further S2′-oriented optimized series with in vivo antithrombotic evaluation [72,73]. These studies reinforce that enzymatic potency alone is insufficient for translational prioritization.

The asundexian/BAY 2433334 program was among the most translationally advanced small-molecule FXIa SAR programs identified in this review. Roehrig et al. describe a protein structure-based de novo design and lead-optimization strategy aimed at developing an orally bioavailable, reversible FXIa inhibitor. During lead modification, the key challenge was to balance FXIa potency and absorption while improving metabolic stability and the cytochrome P450 interaction profile. This program illustrates a central medicinal chemistry principle for FXIa inhibitors: enzymatic potency alone was insufficient, and successful oral development required simultaneous optimization of absorption, metabolic stability, CYP liability, and overall DMPK properties [10].

Fragment-derived inhibitors provided important starting points for active-site SAR. Fjellström et al. identified S1-binding quinolinone-type P1 fragments and expanded them toward the S1′ and S2′ regions, indicating that prime-side extension was important for achieving potent and selective FXIa inhibition [74]. Wei et al. identified 5-phenyl-1H-pyrazole-3-carboxylic acid-derived fragments and developed 5-phenyl-1H-pyrazole-3-carboxamide derivatives as FXIa lead compounds [75]. Together, these studies illustrate how fragment-derived P1 motifs can serve as starting points for active-site optimization through P1′/P2′ or S1′/S2′ extension [74,75].

Several active-site series then refined this subsite logic. Lei et al. developed 2-phenyl-1H-imidazole-5-carboxamide derivatives; compound 44 g showed a Ki of 0.009 μM, more than 400-fold selectivity over thrombin, FVIIa, and FXa, and molecular modeling indicated binding across the S1, S1′, and S2′ pockets [78]. In several later active-site series, selectivity over plasma kallikrein became a major SAR objective. Yao et al. showed that targeting the S2 subsite can improve FXIa selectivity: compound 35 showed FXIa IC50 of 14.2 nM and a plasma kallikrein selectivity index of 1964, and a putative binding model attributed this profile partly to favorable hydrophobic contacts of the S2-directed N-phenylpiperazine moiety [68]. Dai et al. demonstrated a complementary P2′/S2′ strategy using triazole-based benzoic acid fragments. Their representative compound (S)-10h showed FXIa IC50 of 0.38 nM, up to 150-fold selectivity over plasma kallikrein, and up to 100,000-fold selectivity over FXa and thrombin; MD and MM/GBSA analyses linked this profile to electrostatic differences involving residues near the S2′ pocket, particularly the IP-loop [77].

Developability-oriented SAR was particularly evident in the oxopyridine, pyridine N-oxide, and macrocyclic series. In substituted oxopyridine derivatives, hydrophobic P1 fragments were introduced to strengthen S1-pocket interactions and improve selectivity over plasma kallikrein; compound 4c showed FXIa IC50 of 5.64 nM, plasma kallikrein selectivity of 22.7, aPTT ED2× of 10.9 μM, better Caco-2 permeability than 4m and 4q, and oral bioavailability of 37.5% in rats [76]. Xu et al. used pyridine N-oxide-based inhibitors to exploit nonclassical interactions in the P1′ and P2′ regions; compound 3f achieved Ki of 0.17 nM, oral bioavailability in rat, dog, and monkey, and dose-dependent antithrombotic activity in a rabbit arteriovenous shunt model [69]. In macrocyclic FXIa inhibitors, Yang et al. optimized the macrocycle core and P1 linkers to retain potency while reducing clearance and improving oral bioavailability; compound 6f combined potent FXIa inhibition, selectivity against many relevant serine proteases, favorable preclinical PK, and antithrombotic efficacy without prolonging bleeding time at efficacious doses [70].

Hybrid FXa/FXIa inhibitor series expanded the chemical diversity of the field but should be interpreted primarily as early-stage scaffold-exploration studies. Pyrroloquinolinone-based hybrid scaffolds were evaluated through docking-guided selection, in silico analysis, or docking-based rationalization followed by in vitro anticoagulant testing, providing useful scaffold exploration rather than direct evidence for optimized oral FXIa-selective candidates [80,81,82].

Allosteric FXIa inhibitors represented a distinct medicinal chemistry strategy. Boothello et al. used fragment-based design to develop dimeric sulfated quinazolinone-based allosteric inhibitors directed toward heparin-binding-site-related regions [83]. Argade et al. identified monosulfated benzofuran dimers and trimers as allosteric FXIa inhibitors proposed to act through conformational effects near the active site [28]. In a context-only report, Al-Horani et al. described sulfonated non-saccharide molecules as moderate allosteric FXIa inhibitors; inhibitor 16 showed an IC50 of 4.6 μM, selectivity over several serine proteases, and moderate plasma anticoagulant activity [84]. Collectively, the included and contextual allosteric evidence broadened the mechanistic landscape of FXIa inhibition, but these scaffolds generally remained early-stage and showed micromolar rather than subnanomolar potency [28,83,84].

Taken together, the medicinal chemistry evidence supports a convergent optimization strategy in which active-site potency and protease selectivity are integrated with plasma anticoagulant activity, permeability, oral exposure, metabolic stability, clearance, and in vivo efficacy. The later compound-43, F47, and FE12 programs extend this pattern beyond the earlier pyridine N-oxide and macrocyclic series [69,70,71,72,73]. Computational or QSAR models can prioritize chemical space, but their translational value depends on experimental confirmation [79].

#### 3.4.3. In Vitro Pharmacology and Coagulation Assays

In vitro pharmacology studies provided complementary evidence on direct FXIa inhibition and functional anticoagulant effects in plasma. Direct enzymatic inhibition was quantified in biochemical assays mainly as Ki or IC50, whereas plasma-based activity was assessed using study-specific functional readouts such as aPTT prolongation, EC1.5×, ED2×, PT, FXIa activity, FXI clotting activity, or thrombin generation [11,12,75,76,77]. A context-only sulfonated non-saccharide study also reported plasma anticoagulant activity [84]. This distinction is important because biochemical potency and plasma anticoagulant activity are not interchangeable endpoints. Differences between buffer and plasma potency were partly attributed to plasma protein binding, while functional plasma responses varied with species, reagent sensitivity, and the method used to activate coagulation [11,12,85].

The most consistent PD pattern was selective modulation of the intrinsic pathway. Asundexian produced concentration-dependent FXIa inhibition, reduced FXIa activity in human plasma, inhibited thrombin generation after contact activation or low tissue factor triggering, and prolonged aPTT without affecting PT [11]. Milvexian similarly acted as a reversible active-site FXIa inhibitor and predominantly prolonged aPTT, with minimal effects on PT and thrombin time at concentrations producing marked aPTT prolongation, supporting preferential activity in intrinsic-pathway assays rather than broad inhibition of extrinsic or common-pathway coagulation [12]. This pattern was also observed in the rabbit AV shunt model of venous thrombosis (VTM), where milvexian produced dose-dependent ex vivo aPTT prolongation without significant changes in PT or thrombin time [13].

Several medicinal chemistry series reported both biochemical FXIa potency and plasma-based clotting readouts, allowing comparison between direct enzyme inhibition and functional anticoagulant activity. In the 5-phenyl-1H-pyrazole-3-carboxamide series, compound 7za showed FXIa Ki of 90.37 nM and 1.5 × aPTT of 43.33 μM in rabbit plasma [75]. In substituted oxopyridine derivatives, compound 4c combined FXIa IC50 of 5.64 nM with aPTT ED2× of 10.9 μM [76]. In the triazole-based P2′ series, compound (S)-10h showed FXIa IC50 of 0.38 nM, aPTT EC1.5× of 0.55 μM, and PT EC2× >100 μM [77].

Selectivity over plasma kallikrein and related coagulation serine proteases was another important in vitro pharmacology endpoint. S2-subsite-directed compound 35 showed FXIa IC50 of 14.2 nM, high selectivity over plasma kallikrein, and anticoagulant activity through the intrinsic pathway without affecting the extrinsic pathway [68]. Imidazole-carboxamide compound 44 g showed potent competitive FXIa inhibition, with Ki of 9.0 nM, and more than 400-fold selectivity over thrombin, FVIIa, and FXa [78]. In the context-only sulfonated non-saccharide report, inhibitor 16 inhibited FXIa with an IC50 of 4.6 μM and produced moderate plasma anticoagulant activity with preferential aPTT prolongation, but its broader protease selectivity profile was mixed rather than uniformly selective [84].

Clinical PK/PD studies used the same PD readouts, particularly aPTT, FXIa activity, and FXI:C/FXI clotting activity. In first-in-human and multiple-dose studies, asundexian/BAY 2433334 produced dose-dependent aPTT prolongation and FXIa activity inhibition [14,41]. In the Asian phase I program, PK was evaluated in Chinese and Japanese volunteers, whereas PD was assessed in Japanese participants; in the Japanese study, asundexian prolonged aPTT and inhibited FXIa activity without affecting PT, INR, or FXI concentration [42]. ONO-7684 strongly inhibited FXI:C and increased aPTT [17]. SHR2285 showed exposure-related inhibition of FXI activity and aPTT prolongation after single and multiple oral dosing, including during coadministration with antiplatelet therapy [18,29,30]. Milvexian studies also showed exposure- or concentration-related aPTT prolongation [56,57,58]; reduction of FXI clotting activity was reported in the aspirin/clopidogrel interaction study and in Japanese participants [56,57].

Assay interference studies showed that FXIa inhibitors can complicate laboratory interpretation. Asundexian and milvexian caused concentration-dependent and reagent-dependent prolongation of aPTT-based assays [85,87]. A subsequent study confirmed interference in routine and specialized coagulation assays and showed that activated charcoal-based adsorbents can remove asundexian- and milvexian-associated effects [86]. A multicenter assessment across 23 laboratories further documented clinically relevant assay interference from asundexian and milvexian, emphasizing the need to recognize FXI/FXIa inhibitor exposure when interpreting coagulation testing [88]. These findings indicate that aPTT is useful as a PD marker but can also confound diagnostic assays if drug exposure is unrecognized.

Laboratory mitigation and thrombosis-model studies provided additional mechanistic information. Activated prothrombin complex concentrate and recombinant FVIIa corrected selected milvexian-associated anticoagulant readouts in vitro [89]. DOAC-Stop removed asundexian and milvexian from plasma and reversed their effects on coagulation assays [90]. Catheter-initiated thrombin-generation studies examined how milvexian, asundexian, heparins, and other anticoagulants modify contact-pathway-driven coagulation [66,91]. These laboratory data are relevant to assay interpretation and mechanistic management concepts, but they do not establish clinical effectiveness of reversal strategies.

Overall, the available in vitro and plasma-based data support a coherent PD profile in which direct FXIa inhibition preferentially affects intrinsic-pathway or FXI-sensitive assays, particularly aPTT, FXIa activity, and selected thrombin-generation assays [11]. Milvexian data similarly showed predominant aPTT prolongation with limited effects on PT or thrombin time, supporting lower PT sensitivity to FXIa inhibition [12,13]. Ki or IC50 values should be interpreted alongside, rather than as substitutes for, plasma-based anticoagulant readouts [11,75,76,77]. Assay interference and reversal represent distinct translational issues that require separate evaluation [85,86,89].

#### 3.4.4. ADMET and DMPK

ADMET and DMPK data helped distinguish clinically developable oral FXIa inhibitors from compounds that remained mainly biochemical or early pharmacological leads. Across the medicinal chemistry programs, progression beyond enzymatic potency depended on oral exposure, permeability, metabolic stability, clearance, transporter involvement, and CYP-related interaction risk, although the exact DMPK package differed between series [10,69,70].

The asundexian/BAY 2433334 program was among the most extensively documented DMPK-guided optimization programs in the included dataset. During lead optimization, potency was balanced with absorption, metabolic stability, and an improved CYP interaction profile [10]. Human disposition studies characterized metabolism and excretion [43], while dedicated DDI and intrinsic-factor studies evaluated CYP3A/P-gp modulation, age, sex, renal impairment, hemodialysis, and hepatic impairment [44,45]. A four-study bioavailability program showed essentially complete oral bioavailability and no clinically relevant effect of tablet formulation, food, omeprazole, or antacid on exposure [46]. Population pharmacokinetic modeling across 2914 participants identified statistically significant covariates, including body weight, age, sex, CYP3A4 inhibitors, and renal function, but none supported a clinically relevant dose adjustment [48].

Milvexian was supported by a broad clinical DMPK package. First-in-human data showed rapid absorption, approximately dose-proportional exposure from 20 to 200 mg under fasted conditions, a terminal half-life of 8.3–13.8 h after single ascending doses, and renal excretion below 20% [15]. Bioavailability/formulation, rifampin, itraconazole/diltiazem, hepatic-impairment, renal-impairment, antiplatelet coadministration, and ethnicity-specific studies further characterized exposure and variability [56,57,58,59,60,61,62,63]. Model-informed integration of AXIOMATIC data supported selection of milvexian 100 mg twice daily for LIBREXIA-AF, with no observed relationship between bleeding and milvexian exposure in the source model [64]. A dedicated thorough-QT study found no clinically relevant effect on cardiac repolarization at the studied regimens [65].

Renal impairment produced a distinct but modest exposure effect for milvexian. In participants receiving a single oral 60 mg dose, Cmax was similar across renal-function groups, whereas regression modeling estimated AUC∞ increases of approximately 41% at an eGFR of 30 mL/min/1.73 m^2^ and 54% at an eGFR of 15 mL/min/1.73 m^2^ compared with an eGFR of 90 mL/min/1.73 m^2^. Terminal half-life was longer and renal clearance and urinary excretion were lower with moderate or severe renal impairment, while protein binding and concentration-related aPTT prolongation and FXI clotting-activity reduction were broadly similar across groups [63]. By comparison, the asundexian intrinsic-factor program did not show a monotonic increase in unbound exposure with worsening renal function and did not identify a relevant effect of hemodialysis [45]. These data indicate that impaired renal function can modify exposure in a compound-specific manner, but the available clinical studies do not isolate a transporter-specific renal mechanism; renal-function effects therefore should not be attributed to altered P-gp or another transporter without direct mechanistic evidence.

ONO-7684 had a more limited but informative first-in-human clinical PK/PD profile. In this single- and multiple-ascending-dose study, ONO-7684 showed dose-proportional exposure after single dosing, a half-life of 22.1–27.9 h after repeated fed dosing supporting once-daily administration, and a food effect characterized by reduced Cmax and delayed Tmax with limited impact on AUCinf [17].

SHR2285 was supported by three complementary phase I pharmacology reports. After single oral doses of 50–400 mg, median Tmax was 3.0–4.0 h and mean half-life ranged from 7.6 to 15.8 h [18]. A larger SAD/MAD and food-effect study across 25–600 mg single doses and 100–400 mg multiple doses showed rapid absorption, low accumulation, a less-than-dose-proportional increase in exposure, and minimal food effects [30]. Repeated 200 or 300 mg twice-daily administration with aspirin plus clopidogrel or aspirin plus ticagrelor reached steady state by day 6, with mean half-life values of 13.8–14.5 h [29].

Among medicinal chemistry series, ADMET/DMPK information helped distinguish biochemical leads from more translationally mature candidates. The substituted oxopyridine, pyridine N-oxide, and macrocyclic programs linked potency to permeability, microsomal stability, oral bioavailability, and multi-species PK [69,70,76]. Newer optimized compound-43, F47, and FE12 series added orally developable preclinical candidates with in vivo antithrombotic evaluation [71,72,73]. These programs collectively illustrate that discovery-stage advancement depends on a balanced developability profile rather than potency alone.

Overall, the ADMET/DMPK evidence indicates that advancement of oral FXIa inhibitors depended on the integration of potency with oral absorption, metabolic and transporter characterization, clearance control, DDI assessment, cardiac-safety evaluation, population PK, and predictable human exposure. Asundexian now has formulation/food/pH, intrinsic-factor, DDI, disposition, and population-PK support [43,44,45,46,48], while milvexian has formulation, DDI, renal/hepatic, antiplatelet, model-informed dose-selection, and thorough-QT data [56,57,58,59,60,61,62,63,64,65].

#### 3.4.5. In Vivo Thrombosis and Bleeding Models

In vivo thrombosis and bleeding models provided an important preclinical bridge between enzymatic FXIa inhibition, plasma anticoagulant activity, and potential antithrombotic utility. The most informative studies were those that evaluated both thrombus reduction and bleeding liability, because they allowed assessment of whether FXIa inhibition could produce antithrombotic efficacy without proportional impairment of hemostasis [11,12,70]. Rabbit AV shunt studies additionally provided thrombosis efficacy data and ex vivo coagulation readouts, even when formal bleeding models were not always included [11,13].

Asundexian/BAY 2433334 had a broad preclinical in vivo package. In rabbit models, asundexian reduced thrombus weight in FeCl_2_-induced arterial and VTM models and in an arteriovenous shunt model; these experiments included intravenous or oral administration and prevention or intervention settings. These antithrombotic effects were evaluated alongside bleeding models, including ear, gum, and liver-injury bleeding models. Across these models, asundexian reduced thrombus weight without increasing bleeding times or blood loss, either alone or in combination with antiplatelet therapy [11].

Milvexian showed a similar efficacy–bleeding separation in rabbit models. In an electrolytically mediated rabbit carotid arterial thrombosis model, milvexian prevented and treated thrombosis, while rabbit cuticle bleeding experiments showed limited bleeding liability. When combined with aspirin, milvexian did not meaningfully increase bleeding beyond the effect observed with aspirin alone, supporting an efficacy–hemostasis separation in this preclinical setting [12]. In a separate rabbit arteriovenous shunt model of VTM, milvexian produced dose-dependent thrombus reduction and selective ex vivo aPTT prolongation without significant changes in PT or thrombin time [13].

Earlier optimized small-molecule series also provided in vivo proof of concept. Macrocyclic FXIa inhibitor 6f showed antithrombotic efficacy in a rabbit model without prolonging cuticle bleeding time at efficacious doses [70]. Pyridine N-oxide compound 3f demonstrated dose-dependent antithrombotic activity in a rabbit arteriovenous shunt model [69], and S2-subsite-directed compound 35 inhibited FeCl_3_-induced carotid thrombosis with weak bleeding effects [68]. Newer optimized compounds expanded this evidence: compound 43 showed oral antithrombotic activity in mouse and rabbit models with limited bleeding effects, while F47 and FE12 showed additional in vivo antithrombotic activity in rodent models [71,72,73].

Overall, the in vivo evidence supported a recurring preclinical pattern in which optimized FXIa inhibitors reduced thrombus formation in arterial, venous, or AV-shunt models while producing limited effects on bleeding readouts where directly tested. This pattern was documented most clearly for asundexian, milvexian, macrocyclic compound 6f, and compound 43 [11,12,70,71]. Other series contributed supportive in vivo proof of concept but with narrower thrombosis/bleeding packages or less complete developability data [68,69,72,73].

Supplementary Table S8 organizes the rabbit evidence using common descriptive fields rather than treating heterogeneous endpoints as interchangeable. Antithrombotic activity was quantified by thrombus weight, preservation of carotid blood flow, or related model-specific measures, whereas hemostatic effects were assessed using ear, gum, liver-injury, cuticle, or other bleeding endpoints. Compound 43 adds a further paired rabbit efficacy/bleeding example [71]. Numerical normalization was not performed because injury methods, endpoints, doses, exposure confirmation, and bleeding constructs were not commensurable across studies.

### 3.5. Clinical Evidence

#### 3.5.1. Phase I Studies: Safety, PK/PD, and Target Engagement

Phase I studies of oral small-molecule FXIa inhibitors were used to assess whether human target engagement was predictable, pharmacodynamically selective, and compatible with further clinical development. The phase I evidence base included asundexian/BAY 2433334, ONO-7684, milvexian, and SHR2285. For asundexian/BAY 2433334, target engagement was captured mainly by aPTT prolongation and FXIa activity inhibition [14,41,42]. ONO-7684 was characterized by FXI:C inhibition and aPTT prolongation [17]. Milvexian showed exposure- or concentration-related aPTT prolongation across phase I studies [15,56,57,58], with reduction of FXI clotting activity reported in the Japanese study and in the aspirin/clopidogrel coadministration study [56,57]. SHR2285 showed exposure-related FXI activity inhibition and aPTT prolongation across single-dose, SAD/MAD, food-effect, and antiplatelet coadministration studies [18,29,30].

The first-in-human and multiple-dose studies of asundexian/BAY 2433334 showed predictable oral PK and PD target engagement in healthy volunteers [14,41,42]. Additional clinical pharmacology studies characterized DDI and intrinsic-factor effects [44,45], while formulation/food/pH studies supported essentially complete bioavailability without clinically relevant formulation, meal, omeprazole, or antacid effects [46]. A thorough-QT study found no clinically relevant effect of asundexian on cardiac repolarization [47], and population-PK modeling across 2914 participants did not identify a covariate requiring clinically relevant dose adjustment [48].

ONO-7684 provided a separate first-in-human example of oral FXIa inhibition. In a randomized, placebo-controlled SAD/MAD study, ONO-7684 was well tolerated after single and repeated oral dosing. At 250 mg once daily, maximum FXI:C inhibition reached 92% and the maximum aPTT ratio-to-baseline was 2.78, while PT or INR was not significantly affected, supporting an intrinsic-pathway-selective PD profile [17].

SHR2285 was evaluated in three randomized phase I studies. In the first-in-human study, single oral doses of 50–400 mg produced a maximum mean reduction in FXI activity of 24.92–40.00% with synchronized aPTT prolongation, no effect on PT or INR, and no reported bleeding events [18]. In the SAD/MAD and food-effect study, SHR2285 produced exposure-dependent aPTT prolongation and FXI activity reduction; the maximum geometric-mean FXI activity inhibition at steady state was 73.27–87.77% across 100–400 mg, and the compound was generally well tolerated [30]. During six days of coadministration with aspirin plus clopidogrel or aspirin plus ticagrelor, SHR2285 200 or 300 mg twice daily produced mean maximum FXI activity inhibition of 84.8–92.2% and maximum aPTT prolongation of 2.08- to 2.36-fold, without evidence of an increased bleeding risk in healthy volunteers [29].

Milvexian was supported by a broad phase I and clinical pharmacology package. Across first-in-human, ethnicity-specific, antiplatelet coadministration, formulation/bioavailability, DDI, renal- and hepatic-impairment studies, exposure was predictable and aPTT prolongation was concentration- or exposure-related [15,56,57,58,59,60,61,62,63]. Model-informed analyses integrating phase II data supported 100 mg twice daily for LIBREXIA-AF and found no observed exposure–bleeding relationship in the source model [64]. A randomized thorough-QT study in 66 healthy participants found no clinically relevant concentration-QTcF relationship, with the upper confidence bound for placebo-corrected QTcF remaining below 10 ms [65].

Collectively, phase I and clinical pharmacology studies showed predictable human target engagement for oral small-molecule FXIa inhibition. The principal markers were aPTT and FXIa activity for asundexian, FXI:C and aPTT for ONO-7684, aPTT with FXI clotting activity in selected milvexian studies, and aPTT with FXI activity inhibition for SHR2285 [14,15,16,17,18,29,30,41,56,57,58]. Formulation, intrinsic-factor, DDI, population-PK, model-informed dose-selection, and thorough-QT studies broadened the developability evidence for asundexian and milvexian [44,45,46,47,48,59,60,61,62,63,64,65,66]. These data support exposure, tolerability, and target-engagement assessment but do not establish clinical efficacy or a validated PD threshold for clinical benefit.

#### 3.5.2. Indication-Specific Overview and Translational Framework

Clinical efficacy and safety evidence for oral small-molecule FXIa inhibitors was interpreted by indication rather than as a uniform class effect. This approach was necessary because the included trials differed substantially in clinical population, thrombotic mechanism, comparator, background antithrombotic therapy, endpoint structure, and bleeding definition. Table 2 summarizes the indication-specific efficacy and bleeding signals observed in the included randomized outcome studies.

**Figure 2:**
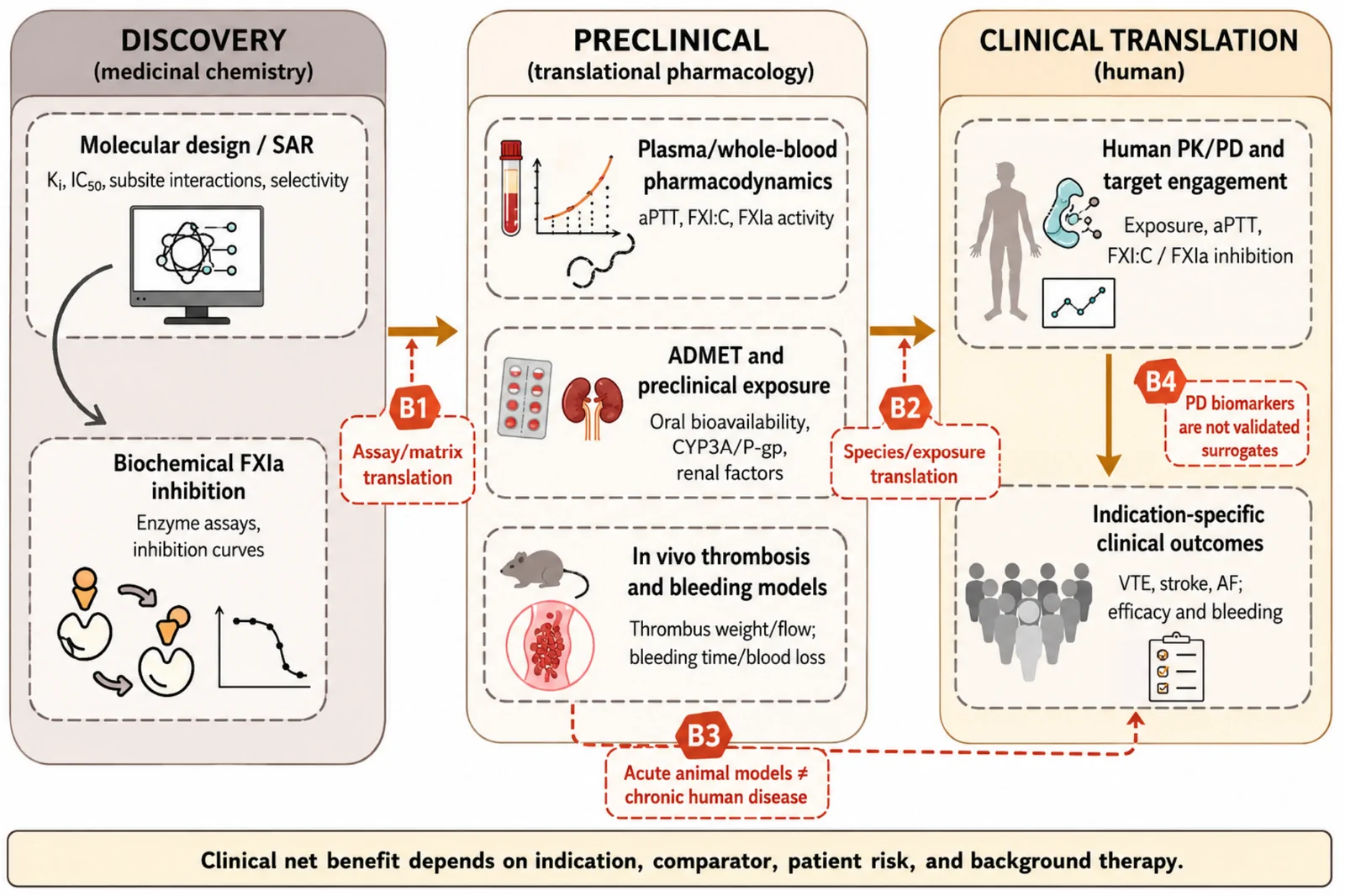
Translational pathway and evidence breakpoints in the development of oral small-molecule FXIa inhibitors. The diagram separates discovery, preclinical translational pharmacology, and human clinical translation and identifies four potential evidence breakpoints: B1, assay/matrix translation; B2, species/exposure translation; B3, limited extrapolation of acute animal thrombosis and bleeding models to chronic human disease; and B4, the absence of validation of PD biomarkers as surrogates for clinical efficacy or bleeding. Net clinical benefit ultimately depends on the indication, comparator, patient risk, and background therapy.

To illustrate how the heterogeneous evidence base was integrated across the review, the included studies were organized into three linked stages: discovery, preclinical translational pharmacology, and human clinical translation. Figure 2 maps four points at which favorable upstream findings may fail to translate downstream: assay/matrix translation (B1), species/exposure translation (B2), extrapolation from acute animal models to chronic human disease (B3), and progression from human PK/PD target engagement to clinical benefit (B4).

In postoperative VTE prophylaxis after knee arthroplasty, milvexian provided a clear phase II clinical efficacy signal. Milvexian reduced postoperative VTE in a dose-related manner compared with enoxaparin, while clinically relevant bleeding remained infrequent and broadly comparable with enoxaparin [31]. This study provided proof of concept that oral FXIa inhibition can translate into measurable antithrombotic efficacy in a selected short-term postoperative VTE setting.

In AF, PACIFIC-AF primarily supported a favorable major/clinically relevant non-major bleeding signal for asundexian compared with apixaban, while thrombotic endpoints were exploratory [49]. OCEANIC-AF subsequently showed that asundexian produced less major bleeding but substantially more stroke or systemic embolism than apixaban [35]. A subgroup analysis suggested that the efficacy deficit differed according to prior oral anticoagulant use but did not alter the overall conclusion that reduced bleeding did not ensure preserved AF stroke prevention [50].

In patients after AMI, PACIFIC-AMI showed strong dose-dependent FXIa inhibition with asundexian on background antiplatelet therapy, but no clear reduction in ischemic outcomes was demonstrated [34]. Bleeding was low and broadly comparable with placebo. Pooled phase II bleeding analyses subsequently found no significant association between asundexian exposure and major/clinically relevant non-major bleeding across the PACIFIC program [51].

In secondary prevention after noncardioembolic ischemic stroke or high-risk TIA, phase II and phase III results differed. PACIFIC-Stroke did not reduce its composite primary endpoint, although bleeding was not increased versus placebo [32]; secondary MRI analyses provided additional information on infarct pattern, cerebral microbleeds, and hemorrhagic transformation without establishing an independent parent-trial efficacy claim [52,53,54]. AXIOMATIC-SSP similarly did not establish a clear dose–response for its primary composite endpoint [33]. In contrast, OCEANIC-STROKE randomized 12,327 patients and showed lower recurrent ischemic stroke with asundexian than placebo on planned antiplatelet therapy (6.2% vs. 8.4%; HR 0.74, 95% CI 0.65–0.84), while major bleeding was 1.9% versus 1.7% (HR 1.10, 95% CI 0.85–1.44) [36].

Overall, the clinical evidence demonstrates that net benefit is indication- and comparator-dependent rather than a uniform class property. Positive efficacy signals are now available in postoperative VTE prophylaxis and phase III secondary prevention after noncardioembolic stroke [31,36]. Conversely, PACIFIC-AMI and the phase II stroke programs were neutral on their primary efficacy endpoints, and OCEANIC-AF showed inferior efficacy versus apixaban despite less major bleeding [32,33,34,35,36].

#### 3.5.3. Randomized Phase II and III Outcome Trials

Randomized efficacy and safety studies of oral small-molecule FXIa inhibitors now span postoperative VTE prophylaxis, noncardioembolic secondary stroke prevention, AMI, and AF. Because these indications differ in thrombotic mechanism, background therapy, comparator, endpoint structure, and bleeding risk, results were interpreted by indication and parent trial rather than generalized as a class effect.

Milvexian showed dose-related efficacy versus enoxaparin after knee arthroplasty [31], whereas AXIOMATIC-SSP did not establish a clear dose–response in secondary stroke prevention [33]. PACIFIC-Stroke and PACIFIC-AMI likewise did not establish significant primary efficacy benefits for asundexian on background antiplatelet therapy [32,34]. OCEANIC-STROKE subsequently demonstrated a phase III reduction in recurrent ischemic stroke with asundexian versus placebo on antiplatelet background therapy [36].

In AF, PACIFIC-AF primarily supported a lower major/clinically relevant non-major bleeding signal for asundexian compared with apixaban, while efficacy endpoints were exploratory [49]. OCEANIC-AF then demonstrated inferior prevention of stroke/systemic embolism versus apixaban despite less major bleeding [35]. The contrast between OCEANIC-AF and OCEANIC-STROKE indicates that successful FXIa inhibition cannot be inferred from target engagement alone and depends on indication, comparator, and background therapy [35,36].

**Figure 3:**
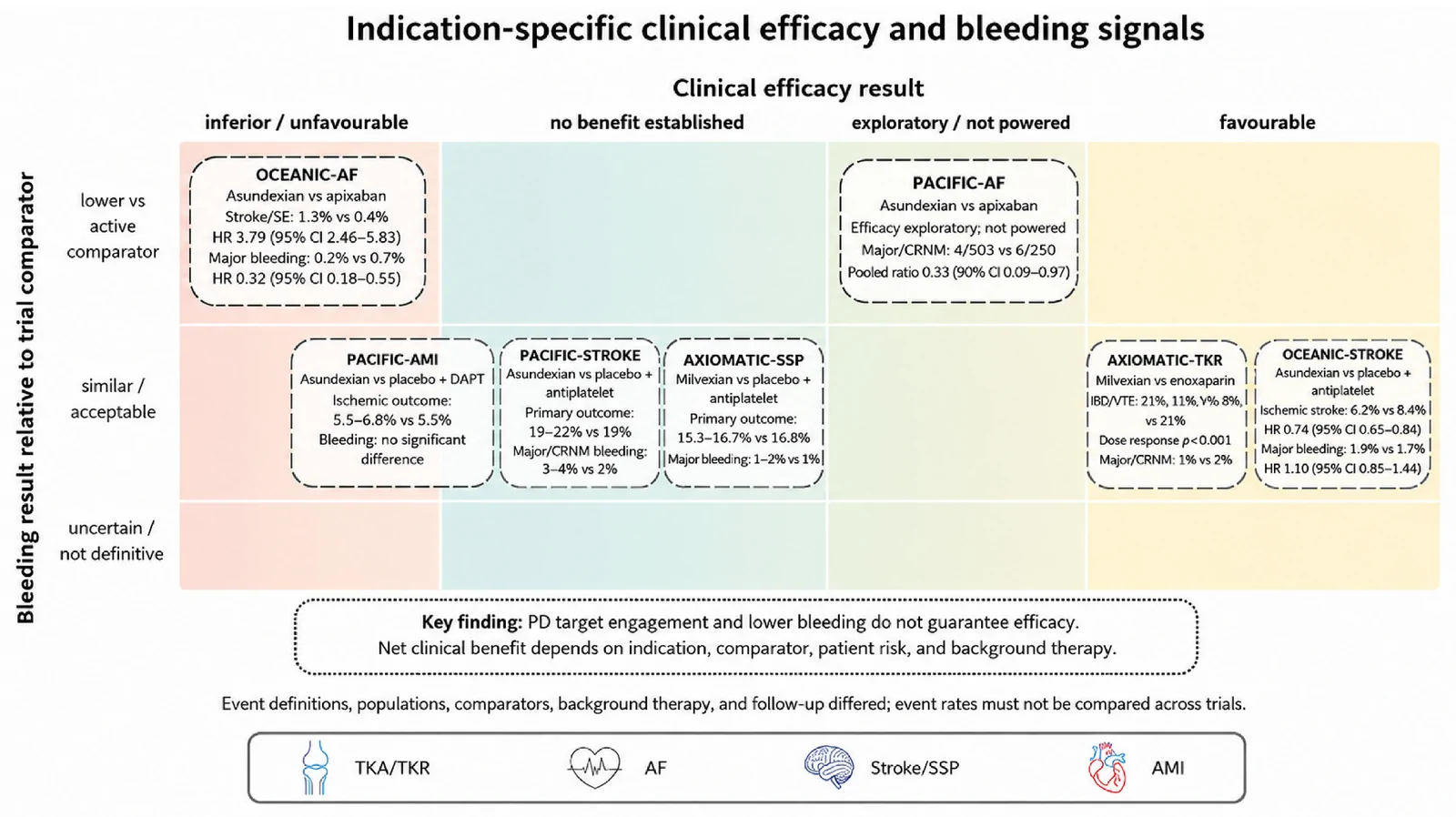
Indication-specific clinical efficacy and bleeding signals from randomized phase II and III trials of oral small-molecule FXIa inhibitors. Each study is positioned according to its principal efficacy result and its bleeding result relative to the trial-specific comparator. The matrix is a conceptual synthesis and does not represent a pooled analysis or a direct comparison among trials. Event definitions, populations, comparators, background therapy, doses, and follow-up differed; event rates and effect estimates should therefore not be compared directly across studies [31,32,33,34,35,36,49].

Figure 3 maps the seven randomized parent outcome trials using their principal quantitative efficacy and bleeding results. The horizontal and vertical positions summarize each trial relative to its own comparator and are not intended as a between-trial ranking. The contrast between OCEANIC-AF and OCEANIC-STROKE illustrates the indication- and comparator-specific nature of clinical translation: asundexian was inferior to apixaban for stroke or systemic embolism prevention in AF despite less major bleeding, whereas it reduced ischemic stroke versus placebo on background antiplatelet therapy after noncardioembolic stroke [35,36].

No core phase II efficacy/safety evidence for oral small-molecule FXIa inhibitors was identified in hemodialysis, catheter-associated thrombosis, or extracorporeal circuit settings. Studies in these areas have largely involved non-oral or biologic FXI/FXIa-targeting strategies, so they should be discussed separately as contextual evidence rather than used to infer efficacy or safety of oral small-molecule FXIa inhibitors.

#### 3.5.4. Pharmacodynamic Markers Across Clinical Studies

Across clinical studies, aPTT, FXIa activity, FXI:C, and FXI clotting activity were useful markers of pathway engagement but were not measured uniformly and should not be treated as interchangeable endpoints. Pooled phase II bleeding analyses found no significant exposure–major/clinically relevant non-major bleeding relationship for asundexian [51], while model-informed milvexian analyses likewise found no observed bleeding–exposure relationship in the dose-selection dataset [64]. These findings do not define a universal safety threshold.

Where assessed, PT or INR generally showed little or no response, supporting PD selectivity for FXI/FXIa-sensitive pathways rather than broad suppression of extrinsic-pathway coagulation. This pattern was reported for asundexian/BAY 2433334 in multiple-dose and Japanese phase I studies [41,42] and for ONO-7684 in its first-in-human SAD/MAD study [17]. Preclinical and translational studies of asundexian and milvexian similarly supported preferential effects on intrinsic-pathway assays, with aPTT being more responsive than PT or thrombin-time-based readouts [11,12,13].

The relationship between exposure and PD response was most clearly documented in phase I studies. Asundexian/BAY 2433334 showed concentration-dependent FXIa activity inhibition and aPTT prolongation [14,41]. ONO-7684 showed exposure-dependent FXI:C inhibition and aPTT prolongation [17]. Milvexian showed exposure- or concentration-related aPTT prolongation, with FXI clotting activity reduction reported in selected studies [15,56,57,58]. These findings indicate that aPTT, FXIa activity, FXI:C, and FXI clotting activity are useful markers of target engagement, but they should not be treated as interchangeable endpoints because each reflects a different assay system and not all were measured for every compound.

Preclinical data provided a mechanistic bridge between direct FXIa inhibition, plasma anticoagulant effects, and in vivo antithrombotic activity. Asundexian inhibited FXIa activity, prolonged aPTT, and reduced thrombin generation in FXI-sensitive experimental systems, while also showing antithrombotic activity in animal thrombosis models without a proportional bleeding signal [11]. The design and preclinical characterization program for asundexian supported the transition from optimized biochemical potency and DMPK properties to human clinical testing [10]. Milvexian showed reversible direct FXIa inhibition, preferential aPTT prolongation, and antithrombotic activity in rabbit thrombosis models, including arterial and arteriovenous shunt models, with limited effects on bleeding readouts where assessed [12,13].

PD target engagement did not translate uniformly into clinical efficacy. Milvexian showed a positive postoperative VTE signal after knee arthroplasty [31]. PACIFIC-Stroke, AXIOMATIC-SSP, and PACIFIC-AMI did not establish significant primary efficacy benefits despite target engagement or acceptable bleeding profiles [32,33,34]. In AF, OCEANIC-AF showed that less major bleeding with asundexian did not compensate for inferior stroke/systemic embolism prevention versus apixaban [35]. Conversely, OCEANIC-STROKE demonstrated reduced recurrent ischemic stroke versus placebo on background antiplatelet therapy [36]. Thus, PD inhibition can support dose development but cannot serve as a validated surrogate for indication-specific clinical benefit.

Laboratory interpretation of FXIa-inhibitor PD also requires caution. Asundexian and milvexian can prolong aPTT-based assays in a concentration- and reagent-dependent manner [85,86,87,88]. Activated charcoal adsorbents, including DOAC-Stop approaches, can remove drug-associated interference in vitro [86,90]. These findings reinforce that aPTT is both a useful PD readout and a potential source of diagnostic assay interference when drug exposure is unrecognized.

Taken together, clinical PD data support coherent target engagement but not outcome predictability. A small mechanistic OCEANIC-AF substudy (*n* = 20) found approximately four-fold higher endogenous thrombin potential with asundexian than with apixaban (760.5 ± 411.7 vs. 199.8 ± 123.0 nM·min; *p* = 0.002) and approximately four-fold higher peak thrombin generation (81.8 ± 55.6 vs. 17.4 ± 11.1 nM; *p* = 0.005) [55]. These measurements were obtained near peak exposure, drug concentrations were not measured, and the analysis was exploratory. The data therefore generate a plausible mechanism for the AF efficacy deficit but do not establish causality or a validated threshold.

Supplementary Table S7 integrates dose/exposure context, principal PD response, and efficacy, bleeding, or safety outcome across the clinical evidence base. High degrees of FXI/FXIa inhibition or marked aPTT prolongation occurred in studies with divergent outcomes, including favorable postoperative VTE efficacy, neutral phase II stroke/AMI results, inferior AF efficacy versus apixaban, and positive phase III secondary stroke efficacy versus placebo [31,32,33,34,35,36]. Because assays, sampling times, populations, comparators, and endpoints differed, no common dose, exposure, aPTT, FXI:C, FXIa activity, or FXI activity threshold for thrombotic protection or bleeding risk could be derived.

#### 3.5.5. Bleeding and Thrombotic Outcomes

Bleeding and thrombotic outcomes were interpreted by indication because randomized trials differed in population, background antithrombotic therapy, comparator, endpoint structure, and bleeding definitions. The evidence now includes phase II postoperative VTE, AF, AMI, and secondary stroke programs as well as phase III OCEANIC-AF and OCEANIC-STROKE [31,32,33,34,35,36,49].

In postoperative VTE prophylaxis after knee arthroplasty, milvexian provided a clear phase II signal of clinical antithrombotic efficacy. Milvexian reduced postoperative VTE in a dose-related manner compared with enoxaparin, met prespecified proof-of-efficacy criteria, and was not associated with major bleeding, while clinically relevant bleeding remained low in this short-term orthopedic setting. These data support clinically measurable antithrombotic efficacy with a favorable bleeding profile in postoperative VTE prophylaxis; however, additional studies are needed before extrapolating these findings to arterial thrombosis indications [31].

In secondary prevention after noncardioembolic ischemic stroke or high-risk TIA, phase II trials did not establish definitive primary efficacy benefits: AXIOMATIC-SSP showed no significant dose–response for its primary composite endpoint [33], and PACIFIC-Stroke did not reduce its composite primary endpoint [32]. Secondary PACIFIC-Stroke analyses provided distinct MRI safety and infarct pattern information [52,53,54]. OCEANIC-STROKE then provided positive phase III evidence: ischemic stroke occurred in 6.2% with asundexian versus 8.4% with placebo (HR 0.74, 95% CI 0.65–0.84), while major bleeding was 1.9% versus 1.7% (HR 1.10, 95% CI 0.85–1.44) [36]. These data replace a uniformly ‘neutral secondary-stroke’ interpretation with a phase- and trial-specific conclusion.

In AMI, PACIFIC-AMI tested asundexian on top of dual antiplatelet therapy after recent MI. Although asundexian produced dose-dependent, near-complete FXIa inhibition at the 50 mg dose, no reduction in ischemic events was observed versus placebo; however, the trial was underpowered to exclude a clinically meaningful benefit. Bleeding was low and not different between asundexian and placebo, but the trial was not designed for definitive efficacy testing and therefore cannot establish an improved benefit–risk balance after AMI [34].

In AF, PACIFIC-AF primarily informed bleeding safety because its primary endpoint was ISTH major or clinically relevant non-major bleeding, whereas thrombotic endpoints were exploratory and underpowered. Asundexian was associated with lower rates of ISTH major or clinically relevant non-major bleeding compared with apixaban, supporting the hypothesis that FXIa inhibition may reduce bleeding relative to apixaban in AF. However, thrombotic endpoints, including ischemic stroke and systemic embolism, were exploratory and underpowered; therefore, the lower bleeding signal in PACIFIC-AF cannot be interpreted as proof of overall net clinical benefit in AF [49]. This limitation was reinforced by OCEANIC-AF, which showed lower bleeding with asundexian but higher rates of stroke or systemic embolism compared with apixaban; the subgroup analysis further examined this finding according to prior oral anticoagulant exposure [35,50]. Therefore, the AF evidence indicates that reduced bleeding with FXIa inhibition does not necessarily imply preserved stroke-prevention efficacy compared with established factor Xa inhibition [35,49,50].

Together, these findings show that the efficacy–bleeding relationship is indication- and comparator-dependent. Positive efficacy evidence now includes postoperative VTE prophylaxis and phase III secondary stroke prevention, whereas PACIFIC-AMI and phase II stroke programs were neutral on primary efficacy endpoints and OCEANIC-AF showed inadequate efficacy versus apixaban despite less major bleeding [31,32,33,34,35,36].

## 4. Discussion

### 4.1. Principal Findings

The principal finding of this review is that oral small-molecule FXIa inhibition is biologically plausible and pharmacologically coherent, but clinical benefit is not a uniform class effect. The evidence base now includes 68 eligible reports spanning medicinal chemistry, computational and laboratory pharmacology, DMPK/PK-PD, animal models, secondary clinical analyses, and seven randomized parent clinical outcome trials. Preclinical programs support the possibility of antithrombotic activity with limited bleeding effects in selected models [11,12,13,70,71], while human studies show reproducible target engagement. The decisive clinical message, however, is indication- and comparator-dependence: OCEANIC-STROKE demonstrated phase III benefit versus placebo on antiplatelet background therapy, whereas OCEANIC-AF showed inadequate efficacy versus apixaban despite less major bleeding [35,36].

Target engagement was reproducibly demonstrated for clinically developed oral small-molecule FXIa inhibitors in phase I and clinical pharmacology studies. Asundexian/BAY 2433334 produced dose- or exposure-related aPTT prolongation and FXIa activity inhibition [14,41,42]. ONO-7684 produced FXI:C inhibition and aPTT prolongation with little effect on PT or INR [17]. Milvexian showed exposure- or concentration-related aPTT prolongation and, in selected studies, reduced FXI clotting activity, including during antiplatelet coadministration [15,56,57,58].

Taken together, the evidence does not support treating oral small-molecule FXIa inhibitors as intrinsically safer or clinically interchangeable alternatives to established anticoagulants. Their clinical value depends on indication biology, dose, magnitude and duration of FXIa inhibition, comparator therapy, and background antithrombotic treatment. Robust PD target engagement and a favorable bleeding signal are therefore insufficient on their own to establish clinical success; therapeutic positioning requires indication-specific evidence of preserved antithrombotic efficacy.

A second major finding is that human PK/PD development is comparatively coherent. Asundexian and milvexian have extensive clinical pharmacology packages covering formulation, food, intrinsic factors, renal/hepatic impairment, DDI, population PK, and cardiac repolarization [44,45,46,47,48,56,57,58,59,60,61,62,63,64,65,66]. These data support predictable exposure and target engagement, but the divergent phase III outcomes confirm that pharmacological coherence cannot be equated with clinical efficacy.

Compared with the narrative review by Prakash et al. [8], the AF-focused systematic review by Xue et al. [37], and the ESKD/hemodialysis-focused systematic review by Steiner et al. [38], this review contributes a narrower oral small-molecule eligibility framework but a broader translational chain. It jointly evaluates candidate design, assay pharmacology, animal efficacy/bleeding models, human exposure and target engagement, and phase II/III outcomes through 30 June 2026, including both OCEANIC-AF and OCEANIC-STROKE. Its principal innovation is therefore the explicit mapping of cross-stage evidence breakpoints and indication-specific boundary conditions, not a claim that all FXI-targeted modalities constitute one evidence class.

### 4.2. Translational Bridge from Preclinical to Clinical Evidence

The translational pathway can be organized around four principal breakpoints, corresponding to those shown in Figure 2. The first translational breakpoint (B1: assay/matrix translation) lies between biochemical FXIa potency and functional anticoagulant activity in plasma or whole blood. Enzyme-level Ki or IC50 values establish direct target inhibition, but functional activity is additionally influenced by protein binding, assay matrix, selectivity, and compound physicochemical properties. Accordingly, successful discovery programs required more than increasing potency: progression depended on balancing FXIa inhibition with selectivity and developability characteristics [9,10,68,69,70].

The second translational breakpoint (B2: species/exposure translation) lies between preclinical developability and human exposure. Cross-species PK, absorption, metabolic stability, clearance, formulation, food effects, renal and hepatic function, and CYP3A/P-gp-mediated interactions can alter exposure even when biochemical potency is unchanged. Asundexian and milvexian clinical pharmacology programs therefore characterized disposition, intrinsic factors, DDIs, formulation effects, and renal or hepatic impairment to define the exposure conditions under which target engagement could be maintained in humans [43,44,45,59,60,61,62,63]. These studies support dose selection, but they do not establish clinical efficacy.

The third translational breakpoint (B3: animal-to-human disease translation) concerns extrapolation from animal thrombosis and bleeding models to human disease. Rabbit FeCl_2_, electrolytic carotid, and arteriovenous shunt models provide controlled proof of antithrombotic activity, sometimes paired with ear, gum, liver-injury, or cuticle bleeding endpoints [11,12,13,70]. They do not reproduce chronic atrial substrate, low-flow left-atrial stasis, comorbidity, platelet and tissue-factor contributions, or background antithrombotic treatment. Positive human efficacy signals are now present in short-term postoperative VTE prophylaxis and phase III noncardioembolic secondary stroke prevention, whereas phase II AMI and stroke programs were neutral and OCEANIC-AF was unfavorable versus apixaban [31,32,33,34,35,36]. Animal efficacy–bleeding separation should therefore be interpreted as model-specific proof of concept, not a predictor of net clinical benefit.

The fourth translational breakpoint (B4: PD biomarker-to-outcome translation) lies between human PD target engagement and clinical outcome benefit. Asundexian, milvexian, ONO-7684, and SHR2285 produced reproducible aPTT, FXIa activity, FXI:C, or FXI-clotting-activity responses in phase I or clinical pharmacology studies [14,15,17,18,29,30,41,42,56,57,58]. However, high levels of target engagement occurred in programs with divergent clinical outcomes: milvexian showed a favorable VTE signal after knee arthroplasty, whereas PACIFIC-Stroke, AXIOMATIC-SSP, and PACIFIC-AMI did not establish primary efficacy benefits, and OCEANIC-AF showed inferior stroke/systemic embolism prevention versus apixaban despite less major bleeding [31,32,33,34,35]. PD markers are therefore valuable for confirming mechanism and informing dose selection, but they are not validated surrogate endpoints for clinical efficacy or bleeding.

Overall, translation is strongest for mechanism-to-target engagement and weakest for target engagement-to-clinical benefit. The principal obstacles are assay-context dependence, cross-species and patient-level differences in exposure, incomplete correspondence between experimental thrombosis models and human disease biology, and the absence of validated PD thresholds that predict hard efficacy and bleeding outcomes. Development should therefore integrate potency and selectivity with DMPK, indication-specific pathophysiology, active-comparator efficacy, and clinically meaningful bleeding endpoints rather than assuming that success at one translational stage guarantees success at the next.

### 4.3. Differences Between Inhibitor Modalities

FXI/FXIa-targeting strategies should not be considered a clinically homogeneous class simply because they target the same coagulation pathway. In this review, the core body of evidence was limited to oral small-molecule FXIa inhibitors and discovery-stage series of small-molecule FXIa inhibitors of sufficient maturity and oral anticoagulant development potential. Non-small-molecule and non-oral FXI/FXIa-targeting strategies were addressed solely for context and were excluded from the efficacy or safety considerations for the individual oral small-molecule programs.

The most important translational breakpoint occurs between human target engagement and indication-specific clinical benefit. OCEANIC-AF demonstrated that lower bleeding and prior FXIa target engagement did not preserve efficacy versus apixaban in AF [35]. OCEANIC-STROKE, by contrast, demonstrated reduced recurrent ischemic stroke versus placebo on background antiplatelet therapy [36]. These findings indicate that the clinically relevant question is not whether FXIa is inhibited, but whether the achieved degree and duration of inhibition are sufficient for the thrombotic mechanism, comparator, and background therapy of a given indication.

These pharmacological properties did not translate into consistent clinical benefit. Milvexian showed a favorable dose–response signal for postoperative VTE prevention [31] but no clear dose-dependent efficacy advantage in AXIOMATIC-SSP [33]. Asundexian phase II programs in AF, AMI, and noncardioembolic ischemic stroke showed strong target engagement and generally acceptable bleeding profiles [32,34,49], yet OCEANIC-AF subsequently demonstrated inadequate efficacy versus apixaban despite less major bleeding [35].

Discovery-stage active-site inhibitor series and optimized preclinical compounds primarily informed potency, selectivity, oral exposure, DMPK optimization, and in vivo antithrombotic activity where available [10,12,13,69,70]. Included allosteric scaffolds and context-only sulfonated small-molecule evidence broadened the mechanistic landscape of FXIa modulation but remained largely exploratory, with supporting evidence derived mainly from enzyme inhibition, in silico modeling, and early anticoagulant assays [28,83,84]. The available data therefore distinguish clinically developed oral FXIa inhibitors from discovery-stage active-site series and exploratory allosteric or sulfonated scaffolds. Clinical value requires not only FXIa inhibition but also robust oral exposure and target engagement [14,15,17,41,42,43,44,45,56,57,58,59,60,61,62,63], favorable DMPK and DDI profiles [10,43,44,45,59,60,61,62,63,68,69,70], and indication-specific efficacy and bleeding outcomes [31,32,33,34,35,49].

### 4.4. Pharmacodynamic Markers and Their Translational Value

PD markers continue to play an important role in the clinical interpretation of oral small-molecule FXIa inhibitors, because they provide evidence of biological engagement of the intrinsic pathway in humans. During phase I and clinical pharmacology studies, aPTT was used most consistently as a marker of pathway engagement. In contrast, more mechanism-proximal assays were more variable on a compound-by-compound basis: FXIa activity for asundexian/BAY 2433334 [14,41,42], FXI:C for ONO-7684 [17], and FXI clotting activity for milvexian in selected studies [56,57]. Exposure- or concentration-related prolongation of aPTT was also observed with milvexian across phase I studies [15,56,57,58]. In general, the lack of effect on PT or INR, when measured, is consistent with preferential modulation of FXI/FXIa-sensitive intrinsic-pathway assays relative to broad suppression of the extrinsic/common pathway of coagulation [15,17,41,42,58]. These data are consistent with preclinical findings for asundexian and milvexian, where aPTT was preferentially affected while PT or thrombin time was generally unaffected when measured [11,12,13]. Nonetheless, preservation of PT or INR should not be taken as evidence of lack of bleeding risk, because routine coagulation assays do not directly measure clinical hemostatic reserve.

One final point is that the interpretation of these biomarkers is dependent on context, both analytical and clinical. The aPTT assay has utility for proof of target engagement, but is neither specific for FXIa inhibition nor a universal standard assay. Laboratory studies with asundexian and milvexian demonstrate that FXIa inhibitor-related interference is assay dependent and reagent dependent, both for laboratory-developed routine assays and for more widely used specialized coagulation assays in a more comprehensive evaluation [85,86]. Activated charcoal-based adsorbents can adsorb FXIa inhibitor-related interference in vitro in selected assays, but this relates to analytical rather than clinical interpretation [86]. Thus, laboratory markers can be useful for detecting a drug effect or assay interference, but are not validated for routine therapeutic monitoring in the same way as the INR is with VKA therapy.

Strong PD activity cannot guarantee clinical efficacy. Positive outcome signals have been observed with milvexian after knee arthroplasty and with asundexian in OCEANIC-STROKE [31,36]. In contrast, PACIFIC-AMI, PACIFIC-Stroke, and AXIOMATIC-SSP were neutral on their primary efficacy analyses, and OCEANIC-AF showed inferior stroke/systemic embolism prevention versus apixaban despite less major bleeding [32,33,34,35]. PD markers should therefore be treated as tools for mechanism confirmation and dose development, not validated surrogate endpoints. Future studies should integrate exposure and PD with hard outcomes, appropriate comparators, standardized bleeding definitions, and standardized laboratory assays [31,32,33,34,35,36,85,86,87,88].

The available evidence does not support a universal PD threshold that predicts thrombotic protection or bleeding. Pooled asundexian phase II analyses did not find a significant exposure–major/clinically relevant non-major bleeding relationship [51], and model-informed milvexian analyses similarly did not identify an exposure–bleeding relationship in the analyzed phase II data [64]. OCEANIC-STROKE further demonstrates that hard clinical outcome evidence, rather than aPTT or FXI/FXIa inhibition alone, must define benefit [36].

### 4.5. Efficacy–Safety Dissociation: Promise and Uncertainty

The primary mechanistic hypothesis driving FXI/FXIa inhibition is the potential for at least a partial dissociation between antithrombotic efficacy and bleeding liability. The theoretical basis for this concept is the specific involvement of FXI/FXIa in contact pathway-initiated coagulation and amplification-associated thrombus propagation, while tissue factor-driven hemostasis is relatively more preserved than with anticoagulants that target core common-pathway components such as thrombin or FXa. This hypothesis is mechanistically appealing for oral small-molecule FXIa inhibitors, but it should not be construed as a foregone clinical conclusion [11,12].

Preclinical data support the biological plausibility of this efficacy–safety dissociation. Asundexian, milvexian, and an optimized orally bioavailable macrocyclic FXIa inhibitor reduced thrombus formation in experimental models with limited effects on bleeding time or blood loss where bleeding endpoints were evaluated [11,12,70]. Additional milvexian AV-shunt data supported antithrombotic activity with selective aPTT prolongation and no significant changes in PT or thrombin time [13]. These models are designed primarily to demonstrate pharmacological proof of concept and bleeding plausibility; they cannot predict net clinical benefit across heterogeneous human thrombotic settings.

Clinical experience now provides both supporting and cautionary evidence for efficacy–safety dissociation. Milvexian showed a favorable phase II postoperative VTE efficacy signal with low bleeding [31]. PACIFIC-AF suggested less major/clinically relevant non-major bleeding than apixaban but was not an efficacy trial [49]. OCEANIC-AF established a key boundary condition: less major bleeding did not compensate for inferior stroke/systemic embolism prevention versus apixaban [35]. Conversely, OCEANIC-STROKE showed that asundexian reduced recurrent ischemic stroke versus placebo on background antiplatelet therapy without a statistically significant increase in major bleeding [36]. The hypothesis therefore remains clinically plausible in selected settings but cannot be generalized across indications or comparators.

Differences across indications are not explained by a simple venous-versus-arterial dichotomy. FXI contributes to thrombin amplification in both settings, but its relative importance varies with the initiating stimulus, contact activation, tissue-factor activity, platelet contribution, local shear and stasis, comparator intensity, and background therapy [4]. AF-related thromboembolism develops in a chronic, low-flow atrial environment in which replacing common-pathway FXa inhibition may require broader suppression of thrombin generation. Consistent with this possibility, the exploratory OCEANIC-AF substudy found markedly higher endogenous and peak thrombin generation with asundexian than with apixaban, although its small size (n = 20), peak-window sampling, and absence of drug-level measurements preclude causal inference [55]. By contrast, OCEANIC-STROKE tested added FXIa inhibition against placebo on antiplatelet therapy and showed benefit [36]. Comparator and background therapy are therefore integral to the pathological boundary of the dissociation hypothesis.

In conclusion, oral FXIa inhibitors remain an attractive concept, but available evidence does not support a class-wide assumption that reduced bleeding automatically yields superior net clinical benefit. Positive efficacy evidence now includes postoperative VTE prophylaxis and phase III secondary prevention after noncardioembolic stroke, while OCEANIC-AF demonstrates that efficacy can be inadequate against an active anticoagulant comparator in AF [31,35,36]. The proposed efficacy–safety dissociation should therefore be interpreted as a conditional therapeutic hypothesis that must be demonstrated in each indication against an appropriate comparator.

### 4.6. Implications for Future Drug Development

Future development of oral FXIa inhibitors should focus on indication-specific net clinical benefit rather than maximal FXIa inhibition. Target engagement is reproducible across phase I and clinical pharmacology programs [14,15,17,18,29,30,41,46,47,48,56,57,58,59,60,61,62,63,64,65,66], but phase III evidence demonstrates markedly different outcomes across settings: asundexian was inferior to apixaban for prevention of stroke or systemic embolism in AF [35], yet reduced recurrent ischemic stroke versus placebo on background antiplatelet therapy in OCEANIC-STROKE [36]. Future programs should therefore define therapeutic windows in relation to disease biology, background therapy, and the efficacy of the indication-specific comparator.

From medicinal chemistry and clinical pharmacology perspectives, development should prioritize balanced, translationally plausible candidates rather than maximal enzyme potency alone. The asundexian program and optimized pyridine-N-oxide, macrocyclic, compound-43, F47, and FE12 series show that FXIa potency and protease selectivity must be advanced together with permeability, oral bioavailability, metabolic stability, PK behavior, clearance, and other DMPK properties [10,69,70,71,72,73]. Predictable human PK/PD should be characterized across formulation and food effects, intrinsic factors, renal and hepatic impairment, antiplatelet coadministration, population-level covariates, cardiac repolarization, and drug–drug interactions [44,45,46,47,48,56,57,58,59,60,61,62,63,64,65,66].

Patient selection is also expected to move to a more mechanism- and risk-driven approach. High-bleeding-risk populations will continue to be relevant targets, but low bleeding liability is only clinically meaningful if the drug also retains efficacy against the current standard of care in that indication [49,50]. Target populations will include the elderly, patients with CKD, and patients who require antiplatelet therapy. Standard PK/PD information (including intrinsic factors, DDIs, and coadministration studies) will not replace hard efficacy and net clinical benefit evidence from indication-specific outcome trials [31,32,33,34,44,45,49,50,56,60,61]. For ACS and secondary stroke prevention in particular, the incremental benefit of additional FXIa inhibition on top of background antiplatelet therapy will need to be evaluated, not in isolation [32,33,34,56].

Extracorporeal circuits remain a rational but unproven future indication for oral FXIa inhibitors; current support is mechanistic and preclinical rather than outcome-trial-based [11,12,13,66,91]. Bleeding management and laboratory interpretation also remain development priorities. In vitro studies show that prohemostatic agents or activated charcoal approaches can mitigate selected laboratory effects of FXIa inhibitors [86,89,90], while multicenter assay data confirm clinically relevant interference with some coagulation tests [87,88]. These findings require clinical validation before they can support routine bleeding management pathways.

Current practice sets a demanding comparator standard. The 2024 ESC atrial fibrillation guideline recommends oral anticoagulation for eligible patients and generally prefers DOACs over vitamin K antagonists, except for mechanical heart valves or moderate-to-severe mitral stenosis [92]. Contemporary CHEST VTE guidance likewise generally favors DOAC-based regimens over vitamin K antagonists for eligible patients, while treatment choice and duration remain sensitive to cancer, antiphospholipid syndrome, renal function, bleeding risk, and the clinical phase of VTE [93]. Oral FXIa inhibitors remain investigational and must therefore demonstrate preserved or superior indication-specific efficacy against current care, not only favorable PD or bleeding signals.

Ideally, the next generation of oral FXIa inhibitors would possess highly selective and reversible FXIa inhibition with predictable oral PK/PD, low DDI liability, and an optimal balance of developability attributes. Most critically, however, they will need robust, indication-specific data demonstrating that the level of FXIa inhibition achieved translates into meaningful clinical utility.

## 5. Limitations

The main limitation of this review is the substantial heterogeneity of the included evidence. The evidence base ranged from medicinal chemistry, structure-based design, computational, biochemical, plasma-based, and animal studies to phase I PK/PD studies and phase II/III clinical trials. Because compounds, assay systems, animal models, clinical populations, comparators, endpoints, and reporting formats differed substantially across studies, quantitative meta-analysis was not appropriate, and findings were synthesized narratively.

Comparability across discovery-stage, in vitro, and preclinical studies was limited by differences in FXIa sources, assay conditions, substrates, plasma matrices, clotting reagents, potency metrics, selectivity panels, ADMET/DMPK reporting, animal species, thrombosis models, dosing schedules, exposure confirmation, and bleeding assessments. Therefore, biochemical Ki or IC50 values, plasma-based anticoagulant readouts, and in vivo antithrombotic effects could not be directly compared across all compounds or interpreted as equivalent measures of translational potential.

Clinical interpretation was limited by heterogeneity in trial phase, study population, clinical indication, background antithrombotic therapy, comparator treatment, dose regimen, follow-up duration, endpoint definitions, and bleeding classifications. Most human studies were phase I or clinical pharmacology studies designed to evaluate exposure, tolerability, and PD target engagement rather than definitive clinical efficacy. Consequently, PD markers such as aPTT, FXIa activity, FXI:C, or FXI clotting activity should be interpreted as evidence of pathway engagement, not as validated surrogate endpoints for clinical benefit.

Evidence identification and reporting also introduced limitations. Searches were restricted to English-language publications. The documented initial search and later update/sensitivity workflow could not be merged at record level because retained exports did not permit reliable cross-deduplication against the original 5024 records. Figure 1 therefore reports the pathways separately. Among 180 unique update records, 16 reports were added and 164 were not added; record-level data were insufficient to reconstruct separate overlap, post-cut-off, title/abstract-exclusion, and full-text exclusion counts for those 164 records. The final publication-eligibility cut-off remained 30 June 2026 to avoid selective post hoc extension. A randomized healthy-participant study of milvexian reversal published on 6 July 2026 consequently falls outside the 68-report synthesis and should be assessed in a future update [94]. Reporting bias and risk of bias due to missing results were not formally assessed.

The review was registered in PROSPERO after pilot work and initial searching/screening had begun. The core review question, eligibility framework, and evidence domains remained focused on oral small-molecule FXIa inhibitors. The initial title/abstract screen and primary extraction were conducted by one reviewer; a second reviewer independently verified the 46 reports included in the documented initial synthesis, all 27 original full-text exclusions, and the key extracted data used in that synthesis, but did not repeat the complete title/abstract screen or independently re-extract every variable. These procedures differed from the registered plan for independent screening and data extraction by at least two reviewers and are reported as protocol deviations. Additional reports identified by the later rerun/sensitivity workflow were screened and extracted using the same predefined framework; independent verification by the second reviewer is not claimed for those additions. Study-level RoB 2 and SYRCLE assessments were performed where applicable, but incomplete reporting in animal studies resulted in predominantly unclear judgments. Certainty of evidence was not formally graded.

Finally, the conclusions of this review apply primarily to oral small-molecule FXIa inhibitors and should not be extrapolated to non-small-molecule FXI/FXIa-targeting modalities, such as monoclonal antibodies, antisense oligonucleotides, small interfering RNA, or other biologic and nucleic-acid-based approaches. Trial design, rationale, and baseline characteristics reports without eligible outcome or pharmacological data were treated only as contextual evidence and were not interpreted as proof of efficacy or safety.

Taken together, these limitations mean that the conclusions should be interpreted as a structured qualitative synthesis of heterogeneous translational evidence rather than as a quantitative estimate of class-level efficacy or safety. Further adequately powered, indication-specific clinical outcome trials are required to determine whether oral small-molecule FXIa inhibitors provide a favorable benefit–risk profile compared with established anticoagulant strategies.

## 6. Conclusions

Oral small-molecule FXIa inhibition is biologically and pharmacologically plausible and produces reproducible target engagement, but clinical benefit is not a uniform class effect. Positive efficacy evidence now includes postoperative VTE prophylaxis with milvexian and phase III secondary prevention after noncardioembolic ischemic stroke with asundexian, whereas phase II AMI and stroke programs were neutral on their primary efficacy endpoints and OCEANIC-AF demonstrated inadequate efficacy versus apixaban despite less bleeding. Human aPTT/FXI/FXIa responses support mechanism and dose development but remain unvalidated surrogates for clinical benefit. Future development should prioritize exposure-informed dose selection, low drug–drug interaction liability, and adequately powered outcome trials against indication-appropriate comparators to determine whether lower bleeding can be achieved without loss of antithrombotic efficacy.

## Figures and Tables

**Table 1:** Translational evidence matrix for oral small-molecule FXIa inhibitors and discovery-stage FXIa inhibitor series.

Compound/Series	Evidence Represented in This Review	Main Translational Contribution	Interpretation for the Review
Asundexian/BAY 2433334	Candidate design and preclinical pharmacology were reported for asundexian [10,11]. Human PK/PD and clinical pharmacology evidence includes first-in-human, multiple-dose, ethnicity, disposition, DDI, intrinsic-factor, formulation/food/pH, thorough-QT, and population-PK studies [14,41,42,43,44,45,46,47,48]. Clinical evidence includes AF, AMI, and noncardioembolic stroke parent trials plus secondary analyses [32,34,35,36,49,50,51,52,53,54,55].	Connects structure-based oral candidate development with preclinical FXIa pharmacology, human target engagement, and indication-specific clinical testing.	Advanced oral clinical candidate. The evidence now includes positive phase III secondary stroke efficacy, but OCEANIC-AF demonstrates strong indication- and comparator-dependence [35,36].
Milvexian/BMS-986177/JNJ-70033093	Discovery/preclinical and rabbit thrombosis-model data were reported [9,12,13]. Human PK/PD and clinical pharmacology evidence includes first-in-human, antiplatelet coadministration, ethnicity, formulation/bioavailability, DDI, renal/hepatic impairment, model-informed dose selection, and thorough-QT studies [15,56,57,58,59,60,61,62,63,64,65]. Additional in vitro mechanistic studies evaluated catheter-induced clotting/thrombin generation and anticoagulant effects in neonatal and pediatric plasma [66,67].Clinical outcome evidence was available in postoperative VTE prophylaxis and secondary stroke prevention [31,33].	Provides a broad translational package linking direct FXIa inhibition, oral exposure, clinical pharmacology, and phase II efficacy/safety testing.	Advanced oral clinical candidate. A positive phase II efficacy signal was observed in postoperative VTE prophylaxis; phase III efficacy remains indication-specific and under evaluation.
ONO-7684	A first-in-human SAD/MAD study reported oral PK, FXI:C inhibition, aPTT prolongation, limited PT/INR effect, and tolerability data [17].	Demonstrates human proof of mechanism for another orally administered small-molecule FXIa inhibitor.	Human phase I oral candidate. Evidence supports target engagement, but no clinical outcome evidence was included.
SHR2285	Three phase I reports evaluated first-in-human single-dose PK/PD, SAD/MAD and food effects, and repeated administration with aspirin plus clopidogrel or ticagrelor [18,29,30].	Adds an independent oral FXIa inhibitor program with quantitative exposure–PD data and antiplatelet coadministration evidence.	Human phase I oral candidate. Evidence supports exposure-related FXI activity inhibition and aPTT prolongation, but no clinical outcome evidence was available.
Optimized preclinical oral/developability-oriented FXIa inhibitor series	S2-subsite-directed inhibitors, pyridine N-oxide inhibitors, and macrocyclic FXIa inhibitors provided potency/selectivity and selected in vivo data [68,69,70]. Additional optimized orally bioavailable series included compound 43, F47, and FE12 [71,72,73].	Shows that translational progression requires more than enzymatic potency, including selectivity, developability, and in vivo antithrombotic assessment.	Optimized preclinical evidence. Translationally informative, including newer in vivo efficacy/bleeding packages, but not supported by human outcome data.
Other active-site discovery series	Fragment-derived, pyrazole-carboxamide, oxopyridine, triazole-based P2′, and imidazole-carboxamide series reported active-site SAR, FXIa inhibition, selectivity, and/or plasma-based readouts [74,75,76,77,78]. A machine-learning QSAR model extended computational screening of FXIa inhibitor chemical space [79].	Defines active-site design principles, including S1/S1′/S2′ or P2′-directed optimization and selectivity over related serine proteases.	Discovery-stage active-site evidence. Useful for scaffold prioritization and SAR interpretation, but generally incomplete as oral development packages.
Hybrid FXa/FXIa scaffold studies	Hybrid pyrroloquinolinone-based studies reported synthesis, docking or in silico evaluation, FXa/FXIa inhibition, and in vitro anticoagulant activity [80,81,82].	Expands chemical diversity and illustrates hybrid FXa/FXIa scaffold exploration.	Exploratory hybrid scaffold evidence. These studies should not be interpreted as optimized oral FXIa-selective programs.
Allosteric and sulfated/sulfonated FXIa inhibitor scaffolds	Monosulfated benzofurans and fragment-derived allosteric inhibitors provided enzyme inhibition, computational, or early anticoagulant evidence within the primary synthesis [28,83]. Sulfonated non-saccharide molecules were retained as related context-only evidence [84].	Broadens the mechanistic landscape of FXIa modulation beyond classical active-site inhibition.	Exploratory nonorthosteric evidence. These scaffolds remain less clinically advanced than oral active-site FXIa inhibitors.
Laboratory assay and reversal/neutralization evidence	Assay interference studies evaluated asundexian and milvexian in routine and specialized coagulation assays [85,86,87,88]. Activated charcoal/DOAC-Stop and in vitro prohemostatic studies addressed laboratory mitigation [89,90], while catheter-triggered thrombin-generation studies provided additional mechanistic evidence [66,91].	Clarifies laboratory interpretation, assay interference, and experimental mitigation of FXIa inhibitor-associated anticoagulant effects.	Supportive laboratory pharmacology. Relevant for assay interpretation and mechanistic management concepts, but not direct clinical efficacy evidence.

Abbreviations: AF, atrial fibrillation; aPTT, activated partial thromboplastin time; DDI, drug–drug interaction; FXI:C, factor XI clotting activity; FXIa, activated factor XIa; PK/PD, pharmacokinetics/pharmacodynamics; PT/INR, prothrombin time/international normalized ratio; SAD/MAD, single- and multiple-ascending dose; SAR, structure–activity relationship; VTE, venous thromboembolism.

**Table 2:** Quantitative indication-specific clinical evidence from randomized oral small-molecule FXIa inhibitor outcome trials.

Indication	Trial and Design/Comparator	Efficacy Result	Bleeding Result	Interpretation
Postoperative VTE after knee arthroplasty	AXIOMATIC-TKR; phase II dose-finding; milvexian vs. enoxaparin	BID VTE: 21%, 11%, 9%, and 8% across 25–200 mg vs. 21%; dose–response *p* < 0.001 [31].	Major/CRNM bleeding 1% vs. 2%; any bleeding 4% vs. 4% [31].	Favorable short-term venous proof of concept; outcome evidence, not a PD threshold.
Atrial fibrillation	PACIFIC-AF; phase II; asundexian vs. apixaban	Efficacy exploratory; trial not powered for stroke prevention [49].	Major/CRNM bleeding: 3/249 (20 mg), 1/254 (50 mg), and 6/250 with apixaban; pooled ratio 0.33 (90% CI 0.09–0.97) [49].	Favorable bleeding signal without definitive efficacy evidence.
Atrial fibrillation	OCEANIC-AF; phase III; asundexian vs. apixaban	Stroke/systemic embolism 1.3% vs. 0.4%; HR 3.79 (95% CI 2.46–5.83) [35].	Major bleeding 0.2% vs. 0.7%; HR 0.32 (95% CI 0.18–0.55) [35].	Inferior efficacy despite less bleeding; active-comparator boundary condition.
Acute myocardial infarction	PACIFIC-AMI; phase II; asundexian vs. placebo on dual antiplatelet therapy	Primary ischemic outcome approximately 5.5–6.8% across doses vs. 5.5%; no significant benefit [34].	No statistically significant difference from placebo [34].	Strong target engagement did not establish incremental benefit.
Noncardioembolic secondary stroke prevention	PACIFIC-Stroke; phase IIb; asundexian vs. placebo on antiplatelet therapy	Primary composite 19%, 22%, and 20% vs. 19% [32].	Major/CRNM bleeding 4%, 3%, and 4% vs. 2% [32].	Neutral phase II primary endpoint.
Secondary stroke prevention	AXIOMATIC-SSP; phase II; milvexian vs. placebo on antiplatelet therapy	Primary composite 15.3–16.7% across doses vs. 16.8%; no significant dose– response [33].	Major bleeding 1–2% vs. 1% [33].	No dose-related efficacy improvement.
Noncardioembolic stroke or high-risk TIA	OCEANIC-STROKE; phase III; asundexian vs. placebo on antiplatelet therapy	Ischemic stroke 6.2% vs. 8.4%; HR 0.74 (95% CI 0.65–0.84) [36].	Major bleeding 1.9% vs. 1.7%; HR 1.10 (95% CI 0.85–1.44) [36].	Positive phase III efficacy; benefit remains indication- and comparator-specific.

Abbreviations: AF, atrial fibrillation; FXIa, activated factor XI; ISTH, International Society on Thrombosis and Haemostasis; TIA, transient ischemic attack; VTE, venous thromboembolism.

## Data Availability

All extracted report-level characteristics and synthesis matrices are provided in Supplementary Tables S6–S8. Search strategies, selection counts, exclusion categories, and evidence-handling rules are documented in Supplementary Tables S2–S4. Additional working extraction files are available from the corresponding author on reasonable request.

## Supplementary Materials

The following supporting information can be downloaded at: https://www.mdpi.com/article/10.3390/ph19081298/s1. Supplementary Table S1: PRISMA 2020 checklist; Supplementary Table S2: Database-specific initial and rerun/sensitivity search strategies; Supplementary Table S3: Full-text exclusion reasons from the documented initial eligibility assessment; Supplementary Table S4: Handling of context-only and excluded evidence; Supplementary Table S5A–S5C: Methodological-quality appraisal framework and study-level risk-of-bias assessments; Supplementary Table S6: Characteristics of the 68 included studies/reports represented in the primary systematic synthesis. Supplementary Table S7: Clinical dose/exposure–pharmacodynamic–outcome matrix for oral small-molecule FXIa inhibitors; Supplementary Table S8: Standardized comparison of rabbit thrombosis and bleeding models.

## Abbreviations

The following abbreviations are used in this manuscript:

ACS	Acute coronary syndrome
AF	Atrial fibrillation
AIS	Acute ischemic stroke
AMI	Acute myocardial infarction
aPCC	Activated prothrombin complex concentrate
aPTT	Activated partial thromboplastin time
ASO	Antisense oligonucleotide
AV	Arteriovenous
BID	Twice daily
CKD	Chronic kidney disease
DDI	Drug–drug interaction
DMPK	Drug metabolism and pharmacokinetics
DOAC	Direct oral anticoagulant
FIX	Factor IX
FVIIa	Activated factor VII
FVIII	Factor VIII
FXa	Activated factor X
FXI	Factor XI
FXI:C	Factor XI clotting activity
FXIa	Activated factor XI
INR	International normalized ratio
MD	Molecular dynamics
MI	Myocardial infarction
OAC	Oral anticoagulation
PD	Pharmacodynamics
PK	Pharmacokinetics
PT	Prothrombin time
SAD	Single ascending dose
SAD/MAD	Single- and multiple-ascending dose
SAR	Structure–activity relationship
TIA	Transient ischemic attack
TT	Thrombin time
VKA	Vitamin K antagonist
VKAs	Vitamin K antagonists
VTE	Venous thromboembolism
VTM	Venous thrombosis model
