# Supplementary material for: Translational Development of Oral FXIa Inhibitors: A Systematic Review of Molecular Design, Pharmacology, and Indication-Specific Clinical Evidence
Source: Pharmaceuticals (Basel). 2026 Aug 17;19(8):1298. doi: 10.3390/ph19081298 (PMC13516291; doi:10.3390/ph19081298)
Supplement: Supplementary file 1 [file pharmaceuticals-19-01298-s001.zip › pharmaceuticals-4492052-supplementary.pdf]

Supplementary Tables for the Systematic Review of Oral Small-Molecule FXIa Inhibitors

These supplementary tables provide the completed PRISMA 2020 checklist and document the initial and rerun/sensitivity search strategies, original full-text exclusion categories, handling of context-only evidence, methodological-quality appraisal, characteristics of the 68 included reports, clinical dose/exposure–pharmacodynamic–outcome relationships, and standardized rabbit thrombosis/bleeding reporting.

Supplementary Table S1. PRISMA 2020 checklist.

Section and topic	Item #	Checklist item	Location where item is reported
**Title – Title**	1	Identify the report as a systematic review.	Title.
**Abstract – Abstract**	2	See the PRISMA 2020 for Abstracts checklist.	Abstract.
**Introduction – Rationale**	3	Describe the rationale for the review in the context of existing knowledge.	Introduction.
**Introduction – Objectives**	4	Provide an explicit statement of the objective(s) or question(s) the review addresses.	Introduction, final paragraph.
**Methods – Eligibility criteria**	5	Specify the inclusion and exclusion criteria for the review and how studies were grouped for the syntheses.	Sections 2.3 and 2.5.
**Methods – Information sources**	6	Specify all databases, registers, websites, organisations, reference lists and other sources searched or consulted to identify studies. Specify the date when each source was last searched or consulted.	Section 2.2; Supplementary Table S2.
**Methods – Search strategy**	7	Present the full search strategies for all databases, registers and websites, including any filters and limits used.	Section 2.2; Supplementary Table S2.
**Methods – Selection process**	8	Specify the methods used to decide whether a study met the inclusion criteria of the review, including how many reviewers screened each record and each report retrieved, whether they worked independently, and, if applicable, details of automation tools used in the process.	Section 2.3. Initial title/abstract screening was performed by M.J.; M.G. independently verified all included reports and all full-text exclusions. Disagreements were resolved by consensus.
**Methods – Data collection process**	9	Specify the methods used to collect data from reports, including how many reviewers collected data from each report, whether they worked independently, any processes for obtaining or confirming data from study investigators, and, if applicable, details of automation tools used in the process.	Section 2.3. Primary extraction was performed by M.J.; M.G. independently checked the key extracted data used in the tables and narrative synthesis. Study authors were not contacted.
**Methods – Data items**	10a	List and define all outcomes for which data were sought. Specify whether all results that were compatible with each outcome domain in each study were sought and, if not, the methods used to decide which results to collect.	Section 2.3.
**Methods – Data items**	10b	List and define all other variables for which data were sought. Describe any assumptions made about any missing or unclear information.	Section 2.3.
**Methods – Study risk of bias assessment**	11	Specify the methods used to assess risk of bias in the included studies, including details of the tool(s) used, how many reviewers assessed each study and whether they worked independently, and, if applicable, details of automation tools used in the process.	Section 2.4; Supplementary Tables S5A-S5C. RoB 2 and SYRCLE assessments were performed independently by M.J. and M.G., with disagreements resolved by consensus; other evidence types were appraised descriptively.
**Methods – Effect measures**	12	Specify for each outcome the effect measure(s) used in the synthesis or presentation of results.	Section 2.5; no quantitative meta-analysis was performed.
**Methods – Synthesis methods**	13a	Describe the processes used to decide which studies were eligible for each synthesis.	Section 2.5.
**Methods – Synthesis methods**	13b	Describe any methods required to prepare the data for presentation or synthesis, such as handling of missing summary statistics or data conversions.	Section 2.5; narrative synthesis.
**Methods – Synthesis methods**	13c	Describe any methods used to tabulate or visually display results of individual studies and syntheses.	Section 2.5; Tables 1–2; Figures 2–3; Supplementary Tables S4–S6. Supplementary Tables S7–S8.
**Methods – Synthesis methods**	13d	Describe any methods used to synthesize results and provide a rationale for the choice(s). If meta-analysis was performed, describe the model(s), methods used to identify and quantify statistical heterogeneity, and software package(s) used.	Section 2.5; narrative synthesis was used because of substantial heterogeneity.

Section and topic	Item #	Checklist item	Location where item is reported
**Methods – Synthesis methods**	13e	Describe any methods used to explore possible causes of heterogeneity among study results.	Not applicable; no statistical investigation of heterogeneity was performed. Sections 2.5 and 5.
**Methods – Synthesis methods**	13f	Describe any sensitivity analyses conducted to assess robustness of the synthesized results.	Not applicable; no sensitivity analyses were performed.
**Methods – Reporting bias assessment**	14	Describe any methods used to assess risk of bias due to missing results in a synthesis.	Section 2.4 states that risk of bias due to missing results was not formally assessed; see Section 5.
**Methods – Certainty assessment**	15	Describe any methods used to assess certainty (or confidence) in the body of evidence for an outcome.	Section 2.4 states that certainty of evidence was not formally assessed; see Section 5.
**Results – Study selection**	16a	Describe the results of the search and selection process, from the number of records identified in the search to the number of studies included in the review, ideally using a flow diagram.	Section 3.1; Figure 1.
**Results – Study selection**	16b	Cite studies that might appear to meet the inclusion criteria, but which were excluded, and explain why they were excluded.	Section 3.1; Supplementary Table S3 reports category-level reasons. A study-level list of original full-text exclusions was not reconstructed from retained files; update-stage exclusion counts were not recoverable.
**Results – Study characteristics**	17	Cite each included study and present its characteristics.	Section 3.2; Supplementary Table S6.
**Results – Risk of bias in studies**	18	Present assessments of risk of bias for each included study.	Section 3.3; Supplementary Tables S5B–S5C provide study-level RoB 2 and SYRCLE judgments. Other evidence domains were appraised descriptively, not assigned individual risk-of-bias grades.
**Results – Results of individual studies**	19	For all outcomes, present, for each study, summary statistics for each group (where appropriate) and an effect estimate and its precision, preferably using structured tables or plots.	Sections 3.4 and 3.5; Tables 1–2. Supplementary Table S7.
**Results – Results of syntheses**	20a	For each synthesis, briefly summarise the characteristics and risk of bias among contributing studies.	Sections 3.2-3.5; Tables 1-2; Supplementary Tables S5A-S6. Supplementary Tables S7–S8.
**Results – Results of syntheses**	20b	Present results of all statistical syntheses conducted. If meta-analysis was performed, present for each the summary estimate and its precision and measures of statistical heterogeneity. If comparing groups, describe the direction of the effect.	Not applicable; no statistical synthesis or meta-analysis was performed. Narrative results are presented in Sections 3.4–3.5.
**Results – Results of syntheses**	20c	Present results of all investigations of possible causes of heterogeneity among study results.	Not applicable; no formal heterogeneity analyses were performed.
**Results – Results of syntheses**	20d	Present results of all sensitivity analyses conducted to assess the robustness of the synthesized results.	Not applicable; no sensitivity analyses were performed.
**Results – Reporting biases**	21	Present assessments of risk of bias due to missing results for each synthesis assessed.	Not formally assessed; reported in Sections 2.4 and 5.
**Results – Certainty of evidence**	22	Present assessments of certainty (or confidence) in the body of evidence for each outcome assessed.	Not formally graded; reported in Sections 2.4 and 5.
**Discussion – Discussion**	23a	Provide a general interpretation of the results in the context of other evidence.	Sections 4.1–4.5.
**Discussion – Discussion**	23b	Discuss any limitations of the evidence included in the review.	Section 5.
**Discussion – Discussion**	23c	Discuss any limitations of the review processes used.	Section 5.
**Discussion – Discussion**	23d	Discuss implications of the results for practice, policy, and future research.	Section 4.6 and Section 6.
**Other information – Registration and protocol**	24a	Provide registration information for the review, including register name and registration number, or state that the review was not registered.	Abstract; Section 2.1. PROSPERO CRD420261356169.
**Other information – Registration and protocol**	24b	Indicate where the review protocol can be accessed, or state that a protocol was not prepared.	Section 2.1; PROSPERO record CRD420261356169.
**Other information – Registration and protocol**	24c	Describe and explain any amendments to information provided at registration or in the protocol.	Sections 2.1, 2.5, and 5.

Section and topic	Item #	Checklist item	Location where item is reported
**Other information – Support**	25	Describe sources of financial or non-financial support for the review, and the role of the funders or sponsors in the review.	Funding statement.
**Other information – Competing interests**	26	Declare any competing interests of review authors.	Conflicts of Interest statement.
**Other information – Availability of data, code and other materials**	27	Report which of the following are publicly available and where they can be found: template data collection forms; data extracted from included studies; data used for all analyses; analytic code; any other materials used in the review.	Data Availability Statement; Supplementary Materials.

Note. Checklist items are reproduced from the PRISMA 2020 statement [39]. "Not applicable" indicates methods or analyses that were not performed because the review used a structured narrative synthesis without meta-analysis.

Supplementary Table S2. Database-specific search strategies

Database/source	Search field / syntax	Search strategy used	Limits or role in the review	Records identified
PubMed/MEDLINE	Title/Abstract fields	(("factor XI"[Title/Abstract] OR "factor XIa"[Title/Abstract] OR FXI[Title/Abstract] OR FXIa[Title/Abstract] OR "activated factor XI"[Title/Abstract]) AND (inhibitor*[Title/Abstract] OR "small molecule"[Title/Abstract] OR "small molecules"[Title/Abstract] OR "small-molecule"[Title/Abstract] OR "small-molecules"[Title/Abstract] OR oral[Title/Abstract] OR "drug design"[Title/Abstract]))	English language; publications through 30 March 2026; no lower date limit.	1544
Scopus	TITLE-ABS-KEY	TITLE-ABS-KEY(("factor XI" OR "factor XIa" OR FXI OR FXIa OR "activated factor XI") AND (inhibitor* OR "small molecule*" OR "small-molecule*" OR oral OR "drug design"))	English language; publications through 30 March 2026; no lower date limit.	1686
Web of Science Core Collection / Science Citation Index	Topic field / TS	TS=(("factor XI" OR "factor XIa" OR FXI OR FXIa OR "activated factor XI") AND (inhibitor* OR "small molecule*" OR "small-molecule*" OR oral OR "drug design"))	English language; publications through 30 March 2026; no lower date limit.	1794
Clinical trial registers	Registry search fields	Search terms combined factor XI, factor XIa, FXIa, asundexian, milvexian, BAY 2433334, BMS-986177, ONO-7684, SHR2285, and factor XIa inhibitor.	Used to identify ongoing, completed, or unpublished clinical studies; records without outcome or eligible pharmacological data were treated as context-only.	Not included in database total

Database/source	Search field / syntax	Search strategy used	Limits or role in the review	Records identified
Reference lists and forward citation searching	Manual citation chasing	Reference lists of included studies and relevant reviews were screened; forward citation searching was used to identify potentially eligible records. The SHR2285 and milvexian clinical-pharmacology programs were included in this citation-searching workflow.	Used as an identification method outside bibliographic database searching; four eligible reports (three SHR2285 phase I reports and one milvexian renal-impairment pharmacokinetic report) were identified and included through the PRISMA 2020 other-methods pathway.	4 eligible reports identified and included via the PRISMA 2020 other-methods pathway
PubMed/MEDLINE – rerun export	Title/Abstract fields	(("factor XI"[Title/Abstract] OR "factor XIa"[Title/Abstract] OR FXI[Title/Abstract] OR FXIa[Title/Abstract] OR "activated factor XI"[Title/Abstract]) AND (inhibitor*[Title/Abstract] OR "small molecule"[Title/Abstract] OR "small molecules"[Title/Abstract] OR "small-molecule"[Title/Abstract] OR "small-molecules"[Title/Abstract] OR oral[Title/Abstract] OR "drug design"[Title/Abstract])) AND ("2026/03/31"[Date - Publication] : "2026/07/31"[Date - Publication]) AND english[Language]	Rerun 11 Aug 2026. Technical export through 31 Jul 2026; final eligibility cut-off 30 Jun 2026 applied after export.	43 raw records
Web of Science Core Collection – rerun export	Topic field / TS	TS=(("factor XI" OR "factor XIa" OR FXI OR FXIa OR "activated factor XI") AND (inhibitor* OR "small molecule*" OR "small-molecule*" OR oral OR "drug design")) AND DOP=2026-03-31/2026-07-31	English. Rerun 11 Aug 2026. Technical export through 31 Jul 2026; final eligibility cut-off 30 Jun 2026 applied after export.	24 raw records
Scopus – rerun export	TITLE-ABS-KEY	TITLE-ABS-KEY(("factor XI" OR "factor XIa" OR FXI OR FXIa OR "activated factor XI") AND (inhibitor* OR "small molecule*" OR "small-molecule*" OR oral OR "drug design")) AND PUBYEAR = 2026	English. All 2026 records exported because exact day-level filtering was not used; final eligibility cut-off 30 Jun 2026 applied after export.	92 raw records
Web of Science – compound-specific sensitivity search	Topic field / TS	TS=(asundexian OR milvexian OR "BAY 2433334" OR "BMS-986177" OR "JNJ-70033093" OR "ONO-7684" OR SHR2285) AND DOP=2025-01-01/2026-07-31	English. Sensitivity search used to detect compound-specific eligible reports, including earlier reports not represented in the original synthesis; final eligibility cut-off 30 Jun 2026.	50 raw records
Scopus – compound-specific sensitivity search	TITLE-ABS-KEY	TITLE-ABS-KEY(asundexian OR milvexian OR "BAY 2433334" OR "BMS-986177" OR "JNJ-70033093" OR "ONO-7684" OR SHR2285)	English; years 2025 and 2026. Sensitivity search used to improve retrieval; final eligibility cut-off 30 Jun 2026.	97 raw records

Note. The documented initial database searches identified 5024 records in total: PubMed/MEDLINE, n = 1544; Scopus, n = 1686; and Web of Science Core Collection/Science Citation Index, n = 1794. The searches were rerun on 11 August 2026, with a final publication-eligibility cut-off of 30 June 2026. The rerun and compound-specific database exports yielded 306 raw records; 126 duplicates were removed within this update/sensitivity batch, leaving 180 unique records for screening. Sixteen reports from this database/sensitivity set were added to the primary synthesis, and six additional eligible reports were

identified by citation/reference-list sensitivity searching across the earlier evidence period. Because retained original exports did not permit reliable record-level cross-deduplication against the original 5024 records, the update pathway is reported separately in Figure 1 rather than retrospectively merged.

Supplementary Table S3. Full-text exclusion reasons after eligibility assessment

Exclusion category	n	Operational definition used during eligibility assessment	Treatment in the review
Review, editorial, commentary, or other non-original article	8	Publication did not provide original quantitative, pharmacological, preclinical, or clinical outcome data eligible for extraction.	Excluded at full-text eligibility assessment; used only for background or citation chasing where relevant.
Non-small-molecule or non-oral FXI/FXIa-targeting modality / outside review scope	5	Publication focused on monoclonal antibodies, antisense oligonucleotides, siRNA/RNA-based therapies, other biologics/nucleic-acid approaches, or non-oral modalities not meeting the predefined intervention scope.	Not counted among the 68 reports in the primary systematic synthesis; used only as contextual comparator evidence where relevant.
Design, rationale, or baseline-only article without eligible outcome or pharmacology data	4	Publication described study rationale, protocol, design, or baseline characteristics but did not provide eligible clinical outcome, PK/PD, pharmacological, or mechanistic data.	Used only to contextualize ongoing or planned clinical development programs.
Not directly relevant to oral FXIa inhibitor pharmacology, efficacy, or safety	4	Publication mentioned FXI/FXIa but did not directly evaluate eligible oral small-molecule FXIa inhibitors or relevant translational endpoints.	Excluded from the primary evidence synthesis.
No extractable eligible quantitative or mechanistic data	3	Publication did not provide extractable numerical, mechanistic, PK/PD, pharmacological, preclinical, or clinical data relevant to FXIa inhibition.	Excluded from the primary evidence synthesis.
Secondary or duplicate report without distinct eligible data	3	Publication duplicated data from another included report and did not provide distinct eligible data relevant to the review question.	Not double-counted; the primary or most data-rich report was used for synthesis.
Total	27	Sum of all full-text exclusions after eligibility assessment.	Corresponds to the full-text exclusion total shown in the PRISMA flow diagram and Results 3.1.

Note. The table reports category-level reasons for the 27 full-text exclusions in the documented initial workflow. A study-level list of those excluded reports was not reconstructed in the retained review files. The later update/sensitivity records were screened under the same eligibility criteria, but stage-specific exclusion counts were not retrospectively assigned because record-level cross-deduplication with the original workflow could not be reconstructed.

Supplementary Table S4. Handling of context-only and excluded evidence.

Record type	Scope/examples	Counting rule	Permitted use and interpretation
Trial-design, rationale, or baseline-only reports	Design, rationale, or baseline-characteristics publications for ongoing or planned clinical programs without eligible outcome or pharmacology data.	Not counted among the 68 primary evidence studies unless distinct eligible outcome, PK/PD, or pharmacology data were provided.	May be cited in Introduction, Methods context, or Discussion/Future directions only; not interpreted as proof of efficacy or safety.
Reviews, editorials, commentaries, and meta-analyses	Narrative reviews, systematic reviews, meta-analyses, editorials, letters, or commentaries, including background clinical reviews [8,37–38].	Not counted as primary evidence.	May be used for background, rationale, or citation chasing only.
Reporting guidelines	PRISMA 2020 and PRISMA-S reporting guidance [39–40].	Not counted as evidence records.	Cited only to document reporting standards and search-reporting guidance.
Non-small-molecule FXI/FXIa modalities	Monoclonal antibodies, antisense oligonucleotides, siRNA/RNA-based agents, biologics, or nucleic-acid strategies, including examples cited in the background [20–26].	Excluded from the primary evidence base.	May be mentioned only as contextual comparator modalities; no efficacy or safety conclusions for oral small-molecule FXIa inhibitors should be drawn from them.
Non-oral small-molecule FXIa agents	Parenteral or otherwise non-oral small-molecule FXI/FXIa agents, including BMS-962212 and frunexian when cited as intravenous comparator/context evidence [16,19].	Excluded from the primary oral evidence base unless oral administration was clearly part of the investigated intervention.	May be mentioned only as context if relevant to route-of-administration distinctions.
SHR2285 oral phase I program	Three oral phase I reports provided first-in-human single-dose, SAD/MAD and food-effect, and antiplatelet-coadministration data for SHR2285 [18,29–30].	Counted as three primary evidence reports within the 68-study/report systematic synthesis.	Included in the clinical pharmacology and phase I synthesis as quantitative PK/PD and safety evidence; not interpreted as clinical outcome efficacy evidence.
Heparin-mimetic or modality-adjacent scaffolds	Synthetic heparin mimetics and related modality-adjacent strategies, including a synthetic heparin mimetic [27], together with sulfonated non-saccharide molecules retained as context-only evidence [84].	Not counted among the 68 primary synthesis rows unless a report was explicitly classified as eligible small-molecule scaffold evidence. Reference [84] was retained as context-only evidence.	May be used for mechanistic context only.
Secondary/subgroup analyses with distinct eligible data	Included as eligible reports when they contributed distinct data relevant to the review question; not treated as independent parent trials.	Examples include the OCEANIC-AF prior-OAC subgroup and PACIFIC-Stroke MRI/bleeding analyses [50–54].	Included report-level evidence; parent-trial risk-of-bias appraisal not duplicated.

Record type	Scope/examples	Counting rule	Permitted use and interpretation
Milvexian renal-impairment clinical pharmacology	Single-dose oral milvexian study in participants with normal renal function and moderate or severe renal impairment [63].	Counted as one primary evidence report within the 68-study/report systematic synthesis.	Included in the clinical pharmacology and special-population synthesis as quantitative renal-function PK/PD and safety evidence; renal impairment was not interpreted as proof of a specific transporter-mediated mechanism.

Note. Context-only records may appear in the reference list if cited, but they were not counted among the 68 reports in the primary systematic synthesis. Secondary/subgroup reports were eligible when they provided distinct data relevant to the review question; they were not counted as independent parent trials when derived from an already represented randomized trial.

Supplementary Table S5A. Methodological-quality appraisal framework by evidence type.

Evidence type	Appraisal approach	Domains considered	Typical strengths in this review	Typical limitations and interpretation safeguards
Randomized clinical outcome trials	Formal study-level assessment using Cochrane RoB 2 for the principal efficacy and/or safety outcome emphasized in the review.	Randomization process; deviations from intended interventions; missing outcome data; outcome measurement; selection of the reported result.	Randomized allocation, prespecified outcomes, comparator groups, masking in most trials, and adjudicated clinical or bleeding outcomes.	Several trials were phase II dose-finding or proof-of-concept studies and were not powered for definitive efficacy conclusions. Risk-of-bias judgments were kept distinct from imprecision and clinical applicability.
Phase I / clinical pharmacology studies	Descriptive methodological appraisal.	Dose design; SAD/MAD structure; PK sampling; PD biomarkers; assay methods; safety reporting; intrinsic-factor or DDI design; sample size; population characteristics.	Detailed PK/PD sampling and target-engagement readouts such as aPTT, FXIa activity, FXI:C, and FXI clotting activity.	Small healthy-volunteer or special-population samples; short exposure; not powered for clinical efficacy or rare safety outcomes.
Animal in vivo studies	Formal study-level assessment using the SYRCLE risk-of-bias tool for in vivo thrombosis and bleeding experiments.	Sequence generation; baseline characteristics; allocation concealment; random housing; investigator and outcome-assessor blinding; random outcome assessment; incomplete outcome data; selective reporting; other bias.	Standardized thrombosis and bleeding models, pharmacodynamic measurements, and numerical outcome reporting in selected studies.	Randomization, allocation concealment, housing, blinding, and prespecified reporting were often insufficiently described. Unclear judgments reflect incomplete reporting rather than demonstrated flaws.

Evidence type	Appraisal approach	Domains considered	Typical strengths in this review	Typical limitations and interpretation safeguards
In vitro pharmacology / plasma-based assays	Descriptive methodological appraisal.	Assay conditions; FXIa source; substrate; plasma/whole-blood system; comparator/control; numerical potency data; replicate reporting; selectivity and coagulation readouts.	Direct mechanistic evidence for FXIa inhibition and intrinsic-pathway assay modulation.	Assay systems and reagents vary; aPTT/FXIa-sensitive assays are target-engagement markers, not validated clinical efficacy surrogates.
Medicinal chemistry / SAR / ADMET-DMPK	Descriptive medicinal-chemistry and developability appraisal.	Chemical structure; analogue series; potency/selectivity consistency; oral developability; permeability; solubility; protein binding; metabolic stability; clearance; bioavailability; CYP/P-gp liabilities.	Useful for identifying scaffold-level SAR and developability determinants.	Endpoints and assay platforms vary across programs; discovery-stage activity does not imply clinical developability.
Computational docking / MD / MMGBSA	Descriptive computational-method appraisal.	Protein structure; ligand/protein preparation; docking protocol; validation; scoring; MD protocol; free-energy method; consistency with experimental potency/selectivity.	Supports binding-mode rationalization and subsite-directed design.	Computational findings were interpreted as supportive only, especially when not experimentally validated.

Note. Randomized clinical outcome trials and animal in vivo studies received formal study-level RoB 2 and SYRCLE assessments, respectively. Phase I, clinical-pharmacology, medicinal-chemistry, in vitro, computational, and ADMET/DMPK evidence was appraised descriptively at the domain level using the framework below; individual risk-of-bias grades were not assigned to those study types.

Supplementary Table S5B. RoB 2 judgments for randomized clinical outcome trials.

Study / outcome assessed	RoB 2 domain judgments	Overall judgment	Basis for judgment
[31] AXIOMATIC-TKR; primary VTE efficacy and principal bleeding safety	Efficacy: D1 Low; D2 Low; D3 Low; D4 Low; D5 Low.<br>Bleeding: D1 Low; D2 Low; D3 Low; D4 Some concerns; D5 Low.	Low (efficacy); Some concerns (bleeding)	Centralized randomization and blinded outcome adjudication supported the efficacy assessment. Assignment to milvexian versus enoxaparin was open-label, which could influence recognition or reporting of bleeding despite central adjudication.
[49] PACIFIC-AF; primary composite bleeding outcome	D1 Low; D2 Low; D3 Low; D4 Low; D5 Low.	Low	Randomized, double-blind, double-dummy design with prespecified outcome definitions and balanced follow-up.
[32] PACIFIC-Stroke; primary composite of covert brain infarction or ischemic stroke	D1 Low; D2 Low; D3 Low; D4 Low; D5 Low.	Low	Central randomization, double blinding, placebo control, prespecified MRI/clinical outcomes, and masked outcome assessment.
[33] AXIOMATIC-SSP; primary efficacy outcome	D1 Low; D2 Low; D3 Low; D4 Low; D5 Low.	Low	Randomized, double-blind, placebo-controlled design with prespecified efficacy and safety outcomes and masked assessment.

Study / outcome assessed	RoB 2 domain judgments	Overall judgment	Basis for judgment
[35] OCEANIC-AF; primary stroke/systemic embolism and major bleeding outcomes	D1 Low; D2 Low; D3 Low; D4 Low; D5 Low.	Low	International double-blind randomized design, prespecified outcomes, protocol availability, and independent safety monitoring. Early termination affected precision and interpretation but did not by itself indicate risk of bias.
[34] PACIFIC-AMI; prespecified bleeding and ischemic outcomes	D1 Low; D2 Low; D3 Low; D4 Low; D5 Low.	Low	Randomized, placebo-controlled, double-blind design with prespecified safety and efficacy outcomes and balanced follow-up.
[36] OCEANIC-STROKE; recurrent ischemic stroke and major bleeding outcomes	D1 Low; D2 Low; D3 Low; D4 Low; D5 Low.	Low	Large randomized, double-blind, placebo-controlled phase III trial with prespecified clinical efficacy and major-bleeding endpoints and blinded outcome assessment.

Note. D1, bias arising from the randomization process; D2, bias due to deviations from intended interventions; D3, bias due to missing outcome data; D4, bias in measurement of the outcome; D5, bias in selection of the reported result. Seven randomized parent clinical outcome trials were appraised. Secondary/subgroup analyses [50–54] derive from already appraised parent trials and are not treated as additional independent RoB 2 units.

Supplementary Table S5C. SYRCLE risk-of-bias judgments for animal in vivo studies.

Study / animal model	SYRCLE domain judgments	Overall judgment	Basis for judgment
[11] Asundexian; rabbit $FeCl_2$ and AV-shunt thrombosis plus ear, gum, and liver-injury bleeding models	A1 U; A2 U; A3 U; A4 U; A5 U; A6 U; A7 U; A8 Low; A9 U; A10 Low.	Unclear	Standardized models and reported group results were strengths, but sequence generation, allocation concealment, housing, and blinding were not reported sufficiently for low-risk judgments.
[12] Milvexian; rabbit carotid arterial thrombosis and cuticle bleeding-time models	A1 U; A2 U; A3 U; A4 U; A5 U; A6 U; A7 U; A8 Low; A9 U; A10 Low.	Unclear	Numerical thrombosis and bleeding outcomes were reported, but key safeguards against selection, performance, and detection bias were insufficiently described.
[13] Milvexian; rabbit AV-shunt venous thrombosis model	A1 U; A2 U; A3 U; A4 U; A5 U; A6 U; A7 U; A8 Low; A9 U; A10 Low.	Unclear	Group sizes and the primary thrombus-weight endpoint were reported, but randomization, concealment, random housing, and blinding were not described.
[68] Compound 35; mouse thrombosis, bleeding, and toxicity experiments	A1 U; A2 U; A3 U; A4 U; A5 U; A6 U; A7 U; A8 U; A9 U; A10 Low.	Unclear	The report provided in vivo antithrombotic and bleeding findings, but the information available did not support confident judgments for most SYRCLE domains.

Study / animal model	SYRCLE domain judgments	Overall judgment	Basis for judgment
[69] Compound 3f; rabbit AV-shunt thrombosis model	A1 U; A2 U; A3 U; A4 U; A5 U; A6 U; A7 U; A8 U; A9 U; A10 Low.	Unclear	Dose-related antithrombotic effects were reported, but allocation, housing, assessor blinding, attrition handling, and prespecified reporting were insufficiently described.
[70] Compound 6f; rabbit thrombosis and cuticle bleeding models	A1 U; A2 U; A3 U; A4 U; A5 U; A6 U; A7 U; A8 U; A9 U; A10 Low.	Unclear	The study linked efficacy with preservation of hemostasis, but methodological reporting was insufficient for low-risk judgments across the principal SYRCLE domains.
[71] Compound 43; mouse and rabbit arterial thrombosis with bleeding assessments	A1 U; A2 U; A3 U; A4 U; A5 U; A6 U; A7 U; A8 U; A9 U; A10 Low.	Unclear	In vivo antithrombotic and bleeding findings were reported, but sequence generation, concealment, housing, blinding, and attrition safeguards were not sufficiently described for low-risk judgments.
[72] FE12; rodent in vivo thrombosis/bleeding experiments	A1 U; A2 U; A3 U; A4 U; A5 U; A6 U; A7 U; A8 U; A9 U; A10 Low.	Unclear	The report included in vivo antithrombotic evaluation, but methodological reporting did not support confident low-risk judgments for most SYRCLE domains.
[73] F47; rodent in vivo thrombosis/bleeding experiments	A1 U; A2 U; A3 U; A4 U; A5 U; A6 U; A7 U; A8 U; A9 U; A10 Low.	Unclear	The report linked optimized FXIa inhibition with in vivo antithrombotic testing; randomization, concealment, housing, and blinding were insufficiently reported for definitive judgments.

Note. A1, sequence generation; A2, baseline characteristics; A3, allocation concealment; A4, random housing; A5, blinding of caregivers/investigators; A6, random outcome assessment; A7, blinding of outcome assessor; A8, incomplete outcome data; A9, selective outcome reporting; A10, other sources of bias. Nine reports containing in vivo thrombosis or bleeding experiments were appraised. U, unclear.

Supplementary Table S6. Characteristics of the included studies/reports represented in the primary systematic synthesis.

No. / Ref(s).	Study / compound	Evidence domain and study type	Main extracted data	Role in synthesis
1. [9]	Milvexian / BMS-986177 discovery program	Medicinal chemistry / SAR / preclinical DMPK	SAR, FXIa potency/selectivity, oral bioavailability, PK, candidate optimization.	Main discovery-to-candidate translational program for milvexian.
2. [10]	Asundexian / BAY 2433334 discovery program	Medicinal chemistry / preclinical DMPK	SAR, oral developability, DMPK, PK/PD-oriented optimization.	Main discovery-to-candidate translational program for asundexian.
3. [11]	Asundexian pharmacological profile	Preclinical / translational pharmacology	FXIa inhibition, aPTT, thrombin generation, thrombosis and bleeding models.	Core preclinical pharmacological profile for asundexian.
4. [12]	Milvexian preclinical pharmacology	In vitro / preclinical in vivo	Reversible FXIa inhibition, aPTT, thrombosis, bleeding.	Core preclinical pharmacology for milvexian.
5. [13]	Milvexian rabbit AV-shunt model	Preclinical in vivo; rabbit AV-shunt thrombosis	Thrombus reduction, ex vivo aPTT/PT/TT.	Venous thrombosis model evidence for milvexian.
6. [14]	Asundexian / BAY 2433334	Phase I / clinical pharmacology; first-in-human SAD	PK, aPTT, FXIa activity, safety.	Human target-engagement and early safety evidence.
7. [15]	Milvexian	Phase I / clinical pharmacology; first-in-human SAD/MAD	PK, aPTT, renal excretion, safety.	Human PK/PD and target-engagement evidence.

No. / Ref(s).	Study / compound	Evidence domain and study type	Main extracted data	Role in synthesis
8. [17]	ONO-7684	Phase I / clinical pharmacology; SAD/MAD	PK, FXI:C, aPTT, PT/INR, safety.	Human proof-of-mechanism for another oral FXIa inhibitor.
9. [18]	SHR2285 / first-in-human single-dose study	Phase I / clinical pharmacology; randomized single-ascending-dose study	Oral PK, FXI activity, aPTT, PT/INR, safety and bleeding observations.	First human PK/PD and target-engagement evidence for SHR2285.
10. [28]	Monosulfated benzofurans	Medicinal chemistry / allosteric inhibition	Enzyme inhibition, selectivity, allosteric mechanism.	Exploratory allosteric/sulfated scaffold evidence.
11. [29]	SHR2285 + aspirin/clopidogrel or aspirin/ticagrelor	Phase I / clinical pharmacology; repeated dosing with antiplatelet coadministration	Steady-state PK, FXI activity inhibition, aPTT, safety and bleeding observations.	Antiplatelet-coadministration evidence relevant to combined antithrombotic settings.
12. [30]	SHR2285 / SAD, MAD and food-effect study	Phase I / clinical pharmacology; randomized SAD/MAD and food-effect study	Oral PK, accumulation, food effect, exposure-related aPTT and FXI activity, safety.	Broad dose–exposure–PD characterization supporting further oral development.
13. [31]	Milvexian / postoperative VTE prophylaxis after knee arthroplasty	Clinical outcome; phase II randomized trial	VTE, dose response, bleeding versus enoxaparin.	Main positive clinical efficacy signal in postoperative VTE prophylaxis.
14. [49]	Asundexian / PACIFIC-AF	Clinical outcome; phase II randomized trial	ISTH major/CRNM bleeding and exploratory thrombotic outcomes.	AF bleeding-safety evidence; not definitive stroke-prevention efficacy evidence.
15. [32]	Asundexian / PACIFIC-Stroke	Clinical outcome; phase IIb randomized trial	Recurrent ischemic stroke, covert infarcts, bleeding.	Secondary stroke prevention evidence.
16. [33]	Milvexian / AXIOMATIC-SSP	Clinical outcome; phase II randomized trial	Efficacy, bleeding, dose response, antiplatelet background.	Secondary stroke prevention evidence; indication-specific efficacy/safety.
17. [35]	Asundexian / OCEANIC-AF	Clinical outcome; phase III randomized trial	Stroke/systemic embolism and major bleeding versus apixaban.	Key AF outcome evidence showing insufficient efficacy despite lower bleeding.
18. [41]	Asundexian / BAY 2433334	Phase I / clinical pharmacology; multiple-dose study	Repeated-dose PK/PD, aPTT, FXIa activity, safety.	Repeated-dose pharmacodynamic validation.
19. [42]	Asundexian	Clinical pharmacology; Chinese/Japanese volunteer study	Ethnicity-specific PK/PD, aPTT, FXIa activity, safety.	Population-specific clinical pharmacology.
20. [43]	Asundexian disposition	DMPK / metabolism; rat and human disposition	Mass balance, metabolism, excretion, bioavailability.	Human metabolism/disposition evidence.
21. [44]	Asundexian clinical DDI	Clinical pharmacology / DDI	Exposure changes with CYP3A/P-gp inhibitors and inducer.	DDI and oral developability evidence.
22. [45]	Asundexian intrinsic-factor study	Clinical pharmacology; intrinsic-factor study	Age, sex, renal impairment, hepatic impairment, PK/PD.	Special-population PK/PD evidence.
23. [34]	Asundexian / PACIFIC-AMI	Clinical outcome; phase II randomized trial	FXIa inhibition, ischemic outcomes, bleeding on antiplatelet therapy.	Post-AMI efficacy/safety evidence; hypothesis-generating.
24. [56]	Milvexian + aspirin/clopidogrel	Clinical pharmacology; antiplatelet coadministration	PK, aPTT, FXI clotting activity, safety.	Coadministration evidence relevant to ACS/stroke settings.
25. [57]	Milvexian	Clinical pharmacology; Japanese volunteer study	PK, aPTT, FXI clotting activity, safety.	Ethnicity-specific PK/PD evidence.
26. [58]	Milvexian	Clinical pharmacology; Chinese adult study	PK, PD, safety, tolerability.	Population-specific PK/PD evidence.
27. [59]	Milvexian formulation / bioavailability	Formulation / bioavailability; microtracer study	Absolute oral bioavailability, food/formulation effects.	Oral formulation and exposure evidence.
28. [60]	Milvexian + rifampin	Clinical pharmacology / DDI; induction study	Reduced exposure, PK/PD effects.	CYP3A/P-gp induction evidence.

No. / Ref(s).	Study / compound	Evidence domain and study type	Main extracted data	Role in synthesis
29. [61]	Milvexian + itraconazole/diltiazem	Clinical pharmacology / DDI; inhibition study	Increased exposure, PK/PD effects.	CYP3A/P-gp inhibition evidence.
30. [62]	Milvexian hepatic impairment	Clinical pharmacology; hepatic impairment	Exposure and safety in mild/moderate hepatic impairment.	Special-population dosing evidence.
31. [63]	Milvexian renal impairment	Clinical pharmacology; renal impairment	Single-dose PK, AUC/Cmax, half-life, renal clearance, aPTT, FXI clotting activity, safety.	Special-population PK/PD evidence defining renal-function effects on exposure without establishing a transporter-specific mechanism.
32. [68]	S2-subsite-directed FXIa inhibitors	Medicinal chemistry / structure-based design / in vivo	IC50, plasma kallikrein selectivity, anticoagulant activity, thrombosis/bleeding data.	S2-subsite selectivity and translational proof-of-concept.
33. [69]	Pyridine N-oxide FXIa inhibitors	Medicinal chemistry / DMPK / in vivo	Ki, oral PK, DMPK, rabbit AV-shunt thrombosis.	Strong preclinical oral-developability package.
34. [70]	Macrocyclic FXIa inhibitor	Medicinal chemistry / DMPK / in vivo	Potency, selectivity, PK, thrombosis, bleeding time.	Optimized preclinical oral FXIa inhibitor evidence.
35. [74]	Fragment-derived FXIa inhibitors	Medicinal chemistry / structure-based design	S1 fragments, structure-aided design, potency/selectivity.	Active-site lead-generation evidence.
36. [75]	5-phenyl-1H-pyrazole-3-carboxamide derivatives	Medicinal chemistry / SAR; fragment-based lead generation	FXIa Ki, aPTT, lead optimization.	Discovery-stage FXIa lead evidence.
37. [76]	Substituted oxopyridine derivatives	Medicinal chemistry / SAR / DMPK	IC50, selectivity, aPTT, permeability, oral bioavailability.	Potency/selectivity plus early oral-developability evidence.
38. [77]	Triazole-based benzoic acid P2-prime fragments	Medicinal chemistry / MD / anticoagulant activity	IC50, selectivity, aPTT, MD/MMGBSA.	P2-prime/S2-prime optimization and selectivity evidence.
39. [78]	2-phenyl-1H-imidazole-5-carboxamide derivatives	Medicinal chemistry / SAR	Ki, selectivity over thrombin/FVIIa/FXa, modeling.	Active-site SAR and selectivity evidence.
40. [80]	Hybrid pyrroloquinolinone FXa/FXIa inhibitors	Medicinal chemistry / docking / in vitro	Docking, synthesis, anticoagulant activity.	Exploratory scaffold-level FXIa-related evidence.
41. [81]	Hybrid tetrahydropyrroloquinolinone FXa/FXIa inhibitors	Medicinal chemistry / in silico / in vitro	Synthesis, docking, FXa/FXIa inhibition, anticoagulant testing.	Exploratory hybrid FXa/FXIa scaffold evidence.
42. [82]	Hybrid arylthiazole-pyrroloquinolinone FXa/FXIa inhibitors	Medicinal chemistry / in vitro / in silico	Synthesis, docking, FXa/FXIa inhibition.	Exploratory hybrid FXa/FXIa scaffold evidence.
43. [83]	Allosteric FXIa inhibitors	Medicinal chemistry / allosteric inhibition	Allosteric inhibition and heparin-binding-site-related mechanisms.	Exploratory allosteric FXIa inhibition evidence.
44. [85]	Asundexian and milvexian assay interference	Laboratory pharmacology; assay interference	aPTT/PT/reagent sensitivity, assay interference.	Laboratory interpretation of FXIa inhibitor exposure.
45. [86]	Asundexian and milvexian assay interference/removal	Laboratory pharmacology; interference/removal	Routine/specialized assays, activated charcoal adsorbents.	Translational laboratory-safety interpretation.
46. [89]	Milvexian reversal / neutralization	In vitro reversal / neutralization	aPCC, rFVIIa, thrombin generation, TEG/aPTT correction.	Reversal/neutralization evidence for milvexian-associated anticoagulation.
47. [50]	OCEANIC-AF prior-OAC subgroup	Secondary clinical analysis; phase III subgroup	Stroke/systemic embolism and bleeding according to prior oral anticoagulant use; efficacy interaction reported.	Distinct subgroup evidence from OCEANIC-AF; not an independent parent trial.

No. / Ref(s).	Study / compound	Evidence domain and study type	Main extracted data	Role in synthesis
48. [46]	Asundexian formulation/food/gastric-pH studies	Clinical pharmacology; bioavailability/formulation	Four studies; absolute bioavailability ~103.9%; formulation, food, and gastric-pH effects not clinically relevant.	Supports robust oral bioavailability and dosing flexibility.
49. [47]	Asundexian thorough-QT study	Clinical pharmacology; cardiac repolarization	Placebo/moxifloxacin-controlled crossover; QTcF confidence bound remained below regulatory concern threshold.	Dedicated cardiac-safety evidence.
50. [51]	Pooled asundexian phase II bleeding analysis	Secondary clinical safety analysis	Pooled PACIFIC-AF/AMI/Stroke bleeding; no significant exposure–major/CRNM bleeding association.	Cross-program bleeding/exposure evidence; not an independent parent trial.
51. [52]	PACIFIC-Stroke infarct-pattern / covert-infarct analysis	Secondary clinical/MRI analysis	Treatment effects evaluated according to baseline infarct pattern and MRI covert infarct outcomes.	Distinct secondary efficacy/imaging evidence.
52. [53]	PACIFIC-Stroke cerebral-microbleed analysis	Secondary clinical/MRI safety analysis	New microbleeds 10.2% with any asundexian vs 10.5% placebo; OR 0.96 (95% CI 0.66–1.41).	Distinct MRI bleeding-safety evidence.
53. [54]	PACIFIC-Stroke hemorrhagic-transformation analysis	Secondary clinical/MRI safety analysis	Hemorrhagic transformation and related imaging risk factors assessed in PACIFIC-Stroke.	Distinct MRI safety evidence.
54. [79]	Machine-learning QSAR model of FXIa inhibitors	Computational / QSAR	Machine-learning regression models built on a curated set of approximately 3,026 FXIa inhibitors for virtual screening/rational design.	Computational discovery evidence; not a substitute for experimental validation.
55. [90]	DOAC-Stop removal of asundexian/milvexian	In vitro laboratory / assay mitigation	Activated-charcoal adsorbent removed FXIa inhibitors and reversed their anticoagulant assay effects.	Assay-interference mitigation evidence.
56. [91]	Catheter-initiated thrombin generation with FXI/FXII inhibitors	In vitro mechanistic pharmacology	Platelet-rich-plasma catheter-triggered thrombin generation compared anticoagulant mechanisms.	Contact-pathway/catheter thrombosis mechanistic evidence.
57. [64]	Milvexian model-informed phase III dose selection	Model-informed clinical pharmacology	Integrated PK/efficacy/bleeding modeling supported 100 mg BID for LIBREXIA-AF; no observed exposure–bleeding relationship.	Dose-selection and exposure-response evidence.
58. [65]	Milvexian thorough-QT study	Clinical pharmacology; cardiac repolarization	Randomized crossover, n=66; upper confidence bound for placebo-corrected QTcF remained <10 ms.	Dedicated cardiac-safety evidence.
59. [66]	Milvexian/heparin catheter-induced clotting and thrombin generation	In vitro mechanistic pharmacology	Milvexian alone and with heparin evaluated in catheter-induced clotting and thrombin-generation systems.	Mechanistic contact-pathway evidence.
60. [48]	Asundexian population PK	Population pharmacokinetics	Population-PK analysis of 2,914 participants; covariates did not support clinically relevant dose adjustment.	Population-level exposure predictability.
61. [87]	Asundexian coagulation-assay impact	In vitro / ex vivo laboratory pharmacology	Concentration-dependent effects across a panel of coagulation assays.	Laboratory interpretation and assay-interference evidence.
62. [36]	OCEANIC-STROKE	Clinical outcome; phase III randomized trial	n=12,327; ischemic stroke 6.2% vs 8.4% (HR 0.74, 95% CI 0.65–0.84); major bleeding 1.9% vs 1.7% (HR 1.10, 95% CI 0.85–1.44).	Positive phase III secondary-stroke evidence; contrasts with OCEANIC-AF.
63. [55]	OCEANIC-AF thrombin-generation analysis	Secondary mechanistic clinical analysis	Exploratory OCEANIC-AF substudy, n=20. Endogenous thrombin potential 760.5±411.7 with asundexian vs 199.8±123.0 nM·min with apixaban (P=0.002); peak thrombin generation 81.8±55.6 vs 17.4±11.1 nM (P=0.005). Drug levels were not measured.	Hypothesis-generating mechanistic evidence for the AF efficacy deficit; small sample and noncausal analysis.
64. [67]	Milvexian in neonatal/paediatric plasma	In vitro age-specific anticoagulant pharmacology	Milvexian anticoagulant effects assessed in healthy neonatal and paediatric samples.	Age-related laboratory pharmacology evidence.

No. / Ref(s).	Study / compound	Evidence domain and study type	Main extracted data	Role in synthesis
65. [71]	Compound 43 optimized FXIa inhibitor	Medicinal chemistry / DMPK / in vivo	Potent, highly selective, orally bioavailable FXIa inhibitor with mouse/rabbit antithrombotic testing and limited bleeding effects.	Optimized discovery-to-preclinical candidate evidence.
66. [72]	FE12 five-membered-heterocycle P2′ series	Medicinal chemistry / in vivo	FE12 FXIa IC50 ~4.4 nM with selectivity/developability and in vivo antithrombotic evaluation.	Optimized preclinical active-site series.
67. [73]	F47 S2′-targeted FXIa series	Medicinal chemistry / in vivo	F47 FXIa IC50 ~2 nM with structure-based S2′ optimization and in vivo antithrombotic evaluation.	Optimized preclinical active-site series.
68. [88]	Multicenter coagulation-assay study	Multicenter laboratory / assay interference	Effects of abelacimab, asundexian, and milvexian evaluated across 23 laboratories and multiple coagulation assays.	Cross-laboratory assay-interpretation evidence for small-molecule FXIa inhibitors.

Note. Reference numbers correspond to the final reference list in the main manuscript. Supplementary Table S6 contains exactly 68 eligible studies/reports represented in the primary systematic synthesis. Secondary/subgroup reports are counted when they contribute distinct eligible data but are not interpreted as independent parent trials.

Supplementary Table S7. Clinical dose/exposure–pharmacodynamic–outcome matrix for oral small-molecule FXIa inhibitors.

**Study / ref(s).**	**Dose or exposure context**	**Principal PD response**	**Efficacy, bleeding, or safety outcome**	**Translational interpretation**
Asundexian phase I [14,41–42]	Single doses 5–150 mg and repeated once-daily dosing; terminal half-life approximately 14.2–17.4 h after single dosing. Asian studies showed dose-proportional exposure without clinically relevant interethnic differences.	Dose/concentration-related FXIa-activity inhibition and aPTT prolongation; no relevant PT/INR effect where assessed.	Generally well tolerated; bleeding time after single dosing was similar to placebo; no clinical efficacy endpoint.	Confirms predictable target engagement, but cannot define an efficacy or bleeding threshold.
Asundexian disposition, DDI, and intrinsic-factor studies [43–45]	Near-complete oral bioavailability and mainly nonrenal clearance. Itraconazole increased AUC by approximately 103% with little change in Cmax; induction reduced exposure. Age, sex, CKD, dialysis, and mild/moderate hepatic impairment produced no clinically meaningful exposure change overall.	aPTT response tracked exposure; maximum aPTT ratio-to-baseline was broadly similar across age, renal, dialysis, and hepatic-function groups.	Single doses were generally well tolerated; no asundexian-related bleeding signal was identified in the intrinsic-factor studies.	Exposure can be modified by CYP3A/P-gp interactions, but renal impairment did not produce a monotonic increase in unbound exposure.
PACIFIC-AF [49]	Asundexian 20 or 50 mg once daily versus apixaban 5 mg twice daily.	FXIa inhibition at trough/peak: 81%/90% with 20 mg and 92%/94% with 50 mg.	ISTH major/CRNM bleeding: 3/249 and 1/254 versus 6/250 with apixaban; pooled incidence-proportion ratio 0.33 (90% CI 0.09–0.97). Thrombotic outcomes were exploratory.	Near-complete inhibition coincided with less bleeding, but the study did not establish stroke-prevention efficacy.
PACIFIC-Stroke [32]	Asundexian 10, 20, or 50 mg once daily versus placebo on antiplatelet therapy.	Dose-dependent FXIa inhibition; higher doses produced stronger target engagement.	Primary composite outcome: 19%, 22%, and 20% versus 19% with placebo. Major/CRNM bleeding: 4%, 3%, and 4% versus 2%.	No common PD threshold predicted the primary ischemic endpoint or bleeding.
PACIFIC-AMI [34]	Asundexian 10, 20, or 50 mg once daily versus placebo after AMI on dual antiplatelet therapy.	Dose-dependent FXIa inhibition; greater than 90% reduction at 50 mg.	Primary ischemic outcome was approximately 5.5%–6.8% across asundexian doses versus 5.5% with placebo; bleeding did not differ significantly from placebo.	Strong target engagement did not establish incremental ischemic benefit in this phase II setting.
OCEANIC-AF and prior-OAC subgroup [35,50]	Asundexian 50 mg once daily versus standard-dose apixaban.	Dose selected from earlier PK/PD program; the outcome report did not establish a validated biomarker cutoff.	Stroke/systemic embolism: 1.3% versus 0.4% (HR 3.79, 95% CI 2.46–5.83). Major bleeding: 0.2% versus 0.7% (HR 0.32, 95% CI 0.18–0.55).	Less major bleeding did not compensate for inferior efficacy; prior target engagement was not a surrogate for AF protection.
ONO-7684 phase I [17]	Single doses 1–300 mg and repeated doses up to 250 mg once daily.	At 250 mg once daily, maximum FXI:C inhibition reached 92% and maximum aPTT ratio-to-baseline was 2.78; PT/INR was not significantly affected.	Generally well tolerated; no clinical outcome endpoint and no signal suggesting increased bleeding risk.	Demonstrates strong, pathway-selective PD activity without outcome validation.
SHR2285 first-in-human [18]	Single oral doses 50–400 mg; half-life approximately 7.6–15.8 h.	Maximum mean FXI-activity reduction 24.92%–40.00% with synchronized aPTT prolongation; no PT/INR effect.	No bleeding events were reported; no clinical efficacy endpoint.	Early target engagement was measurable but substantially below the inhibition achieved in later repeated-dose studies.
SHR2285 SAD/MAD and food-effect study [30]	Single doses 25–600 mg and repeated doses 100–400 mg; minimal food effect and low accumulation.	Maximum geometric-mean FXI-activity inhibition at steady state 73.27%–87.77%; exposure-dependent aPTT prolongation.	Generally safe and well tolerated in healthy volunteers; no clinical efficacy endpoint.	Provides a broad dose–exposure–PD relationship but no validated outcome threshold.
SHR2285 with antiplatelet therapy [29]	200 or 300 mg twice daily for 6 days with aspirin/clopidogrel or aspirin/ticagrelor.	Maximum FXI-activity inhibition 84.8%–92.2%; maximum aPTT prolongation 2.08- to 2.36-fold.	All adverse events were mild and no increased bleeding risk was identified in 52 healthy participants.	High PD intensity remained tolerable in a short healthy-volunteer study, but cannot predict clinical bleeding on chronic therapy.

**Study / ref(s).**	**Dose or exposure context**	**Principal PD response**	**Efficacy, bleeding, or safety outcome**	**Translational interpretation**
Milvexian phase I and Asian studies [15,57–58]	Single and multiple doses up to 500 mg; half-life generally approximately 8–14 h. Chinese cohorts received 25–200 mg regimens.	Concentration- or exposure-related aPTT prolongation; FXI clotting activity decreased in selected studies.	Generally well tolerated, with no clinically significant bleeding signal in healthy participants.	Supports reproducible target engagement across populations but not a clinical efficacy threshold.
Milvexian with aspirin and/or clopidogrel [56]	Milvexian 200 mg twice daily for 5 days with aspirin and/or clopidogrel.	Peak and total exposure were similar with and without antiplatelets; exposure-dependent aPTT prolongation and FXI-clotting-activity reduction were preserved.	Eight mild bleeding adverse events occurred in 5 of 113 participants across treatment arms.	Antiplatelets did not materially change milvexian PK/PD in healthy volunteers; the study was not powered for clinical bleeding.
Milvexian formulation, DDI, and hepatic-impairment studies [59–62]	Repeated rifampin reduced Cmax to 22% and AUC to 15% of milvexian-alone exposure. Itraconazole increased AUC 2.5-fold; diltiazem increased AUC by 38%. Mild/moderate hepatic impairment produced broadly similar exposure.	aPTT prolongation remained concentration-related despite exposure modification.	Treatments were generally well tolerated; no serious bleeding signal was identified in these short studies.	CYP3A/P-gp modulation can markedly alter exposure; PD response follows concentration, but outcome consequences remain unvalidated.
Milvexian renal-impairment study [63]	Single oral 60 mg dose in participants with normal renal function and moderate or severe renal impairment (n = 8 per group). Modeled AUC∞ was approximately 41% and 54% higher at eGFR 30 and 15 mL/min/1.73 m2, respectively, than at eGFR 90; Cmax was similar.	Concentration-dependent aPTT prolongation and FXI-clotting-activity reduction were similar across renal-function groups; protein binding was also similar.	Generally safe and well tolerated; no bleeding events were reported in the 24 treated participants.	Renal impairment modestly increased exposure and reduced renal clearance/urinary excretion, but the study did not establish a transporter-specific mechanism.
AXIOMATIC-TKR [31]	Milvexian 25–200 mg once or twice daily versus enoxaparin after knee arthroplasty.	Dose/exposure response was supported by phase I data; the clinical trial used VTE rather than a PD marker as the efficacy endpoint.	VTE with twice-daily milvexian: 21%, 11%, 9%, and 8% across 25–200 mg versus 21% with enoxaparin; dose response P<0.001. Any bleeding 4% versus 4%; major/CRNM bleeding 1% versus 2%.	The clearest positive efficacy signal was established from clinical events, not from a biomarker cutoff.
AXIOMATIC-SSP [33]	Milvexian 25 mg once daily to 200 mg twice daily versus placebo on dual antiplatelet therapy.	Clinical doses were selected from the PK/PD program; no validated aPTT or FXI-activity threshold was prespecified as a surrogate.	Primary composite estimate 15.3%–16.7% across milvexian doses versus 16.8% with placebo; no significant dose response. Major bleeding was 1%–2% versus 1%.	Dose escalation did not produce a corresponding improvement in the composite ischemic endpoint.
Asundexian pooled phase II bleeding [51]	PACIFIC-AF/AMI/Stroke pooled exposure and bleeding analyses.	Asundexian exposure examined against ISTH major/CRNM bleeding.	No significant exposure–major/CRNM bleeding association.	Supports absence of a validated bleeding-exposure threshold in phase II data.
Milvexian model-informed dose selection [64]	Integrated AXIOMATIC PK/efficacy/bleeding modeling; selected 100 mg BID for LIBREXIA-AF.	Model-based exposure/efficacy framework; no observed exposure–bleeding relationship.	Regimen selected for phase III evaluation; not itself an outcome trial.	Illustrates exposure-informed dose selection while preserving need for hard outcomes.
Asundexian formulation / population PK [46,48]	Formulation/food/pH studies and population PK across 2,914 participants.	Exposure variability characterized rather than a single efficacy PD threshold.	No clinically relevant formulation/food/pH effect; no covariate requiring dose adjustment.	Supports exposure predictability but does not validate a clinical surrogate.
Asundexian thorough-QT [47]	Randomized thorough-QT study in healthy adults.	No clinically relevant concentration–QTcF relationship.	No clinically relevant cardiac-repolarization effect.	Dedicated safety evidence outside thrombotic efficacy.
Milvexian thorough-QT [65]	Milvexian 100 or 200 mg every 12 h in randomized crossover study (n=66).	No apparent concentration–QTcF relationship; upper confidence bound <10 ms.	Regimens were generally well tolerated; no clinically relevant QT prolongation.	Dedicated cardiac-repolarization safety evidence.

Study / ref(s).	Dose or exposure context	Principal PD response	Efficacy, bleeding, or safety outcome	Translational interpretation
OCEANIC-STROKE [36]	Asundexian 50 mg once daily versus placebo in 12,327 patients with recent noncardioembolic ischemic stroke/high-risk TIA on antiplatelet therapy.	Dose derived from prior PK/PD program; no validated PD surrogate threshold used for efficacy.	Ischemic stroke 6.2% vs 8.4% (HR 0.74, 95% CI 0.65–0.84); major bleeding 1.9% vs 1.7% (HR 1.10, 95% CI 0.85–1.44).	Positive phase III efficacy versus placebo contrasts with OCEANIC-AF versus apixaban and reinforces indication/comparator dependence.
OCEANIC-AF thrombin-generation substudy [55]	n=20; samples approximately 3 h after dosing following at least 2 weeks of randomized treatment; drug concentrations were not measured.	Endogenous thrombin potential 760.5±411.7 vs 199.8±123.0 nM·min (P=0.002), and peak thrombin generation 81.8±55.6 vs 17.4±11.1 nM (P=0.005), for asundexian vs apixaban.	Mechanistic substudy only; not powered for clinical outcomes. OCEANIC-AF parent outcome remained inferior for stroke/systemic embolism with less major bleeding [35].	Approximately four-fold higher thrombin generation offers a plausible but unproven explanation; the small sample, peak-window sampling, and absent drug levels preclude a threshold or causal claim.

Note. The matrix represents clinical and clinical-pharmacology reports most informative for dose/exposure–PD–outcome interpretation, with closely related evidence grouped by compound and purpose. Quantitative values are study-specific and are not directly comparable across trials. PD markers are interpreted as target-engagement measures, not validated surrogate endpoints for clinical efficacy or bleeding risk.

Supplementary Table S8. Standardized comparison of rabbit thrombosis and bleeding models.

Study / compound	Thrombosis model and endpoint	Bleeding endpoint	Route, regimen, and timing	Comparator context	Exposure / PD confirmation	Interpretive limit
[11] Asundexian	Rabbit $FeCl_2$ arterial and jugular-vein thrombosis and AV shunt; prevention/intervention; thrombus weight.	Ear and gum bleeding time; liver-injury blood loss with serial preweighed swabs.	Intravenous or oral dosing; prevention and intervention schedules varied by experiment.	Vehicle; rivaroxaban; dabigatran; antiplatelet therapy; apixaban combinations.	Plasma exposure and intrinsic-pathway PD were assessed in the source program; group size and exact sampling varied by experiment.	Broad paired package, but injury sites, units, and designs are not numerically interchangeable.
[12] Milvexian	Electrolytic rabbit carotid thrombosis; prevention/treatment; carotid flow and thrombus weight.	Rabbit cuticle bleeding time, including milvexian plus aspirin vs aspirin alone.	Intravenous bolus plus infusion; preventive and therapeutic schedules.	Vehicle and antiplatelet-combination comparisons.	Plasma concentrations and ex vivo aPTT linked exposure to effect; exact timing was experiment-specific.	Acute arterial model does not reproduce chronic human thrombotic disease.
[13] Milvexian	Rabbit AV-shunt venous thrombosis; thrombus weight reduced by approximately 34%, 52%, and 67% across increasing regimens.	No concurrent experimental bleeding endpoint in this report.	Intravenous bolus plus infusion; increasing dose/exposure regimens.	Vehicle; contextual apixaban/dabigatran comparison.	Ex vivo aPTT, PT, and thrombin time; exposure-supported dose response.	Cannot independently establish efficacy–bleeding dissociation.
[69] Pyridine N-oxide 3f	Rabbit AV-shunt thrombosis; dose-related thrombus formation/weight reduction.	No paired bleeding model reported.	Oral/developability-oriented preclinical dosing; study-specific regimen.	Vehicle.	Program reported PK/developability support; extracted review data do not provide a uniform exposure threshold.	In vivo efficacy without direct hemostasis comparison.
[70] Macrocyclic 6f	Electrolytic rabbit carotid thrombosis; antithrombotic efficacy at exposure-supporting doses.	Rabbit cuticle bleeding time, including aspirin context; no prolongation at efficacious doses.	Preclinical oral/developability program; study-specific timing.	Vehicle and aspirin-context comparisons.	Exposure and PD supported efficacious dosing in the source report.	Cuticle bleeding is not interchangeable with ear, gum, or liver blood loss.
[71] Compound 43	$FeCl_3$ rabbit arterial thrombosis; mouse arterial thrombosis; comparator testing with asundexian.	Mouse and rabbit bleeding assessments; limited effects reported.	Oral dosing in an optimized discovery program; study-specific schedule.	Vehicle and asundexian comparator context.	Oral bioavailability and in vivo pharmacology supported candidate selection.	Paired evidence remains model-specific and not predictive of clinical net benefit.

Note. The table standardizes model, endpoint, route/regimen, timing, comparator, and exposure/PD fields; it does not normalize numerical outcome scales. $FeCl_2$/$FeCl_3$ injury, electrolytic carotid thrombosis, and AV-shunt thrombosis are non-equivalent, and bleeding time, blood loss, flow, and thrombus weight measure different constructs. “Study-specific” indicates that exact regimens or group sizes were not consistently available in the review extraction and were not inferred.

Abbreviations

ADMET, absorption, distribution, metabolism, excretion, and toxicity; aPCC, activated prothrombin complex concentrate; aPTT, activated partial thromboplastin time; CRNM, clinically relevant non-major; DDI, drug-drug interaction; DMPK, drug metabolism and pharmacokinetics; FXI:C, factor XI clotting activity; FXIa, activated factor XIa; INR, international normalized ratio; ISTH, International Society on Thrombosis and Haemostasis; MD, molecular dynamics; MMGBSA, molecular mechanics/generalized Born surface area; OAC, oral anticoagulant; PD, pharmacodynamics; PK, pharmacokinetics; PT, prothrombin time; RoB 2, Cochrane Risk of Bias 2; SAD/MAD, single- and multiple-ascending dose; SAR, structure-activity relationship; SYRCLE, Systematic Review Centre for Laboratory Animal Experimentation; TEG, thromboelastography; TT, thrombin time; VTE, venous thromboembolism.
